# ‘Traceless’ tracing of proteins – high-affinity trans-splicing directed by a minimal interaction pair†
†The German Research Foundation (EXC115 Cluster of Excellence – Macromolecular Complexes to R. W. and R. T., SPP 1623 to R. T.) supported the work.
‡
‡Electronic supplementary information (ESI) available. See DOI: 10.1039/c5sc02936h


**DOI:** 10.1039/c5sc02936h

**Published:** 2015-12-21

**Authors:** M. Braner, A. Kollmannsperger, R. Wieneke, R. Tampé

**Affiliations:** a Institute of Biochemistry, Biocenter, and Cluster of Excellence – Macromolecular Complexes , Goethe-University Frankfurt , Max-von-Laue-Str. 9 , 60438 Frankfurt/M. , Germany . Email: tampe@em.uni-frankfurt.de

## Abstract

Protein trans-splicing mediated by split inteins is a powerful technique for site-specific protein modification. Despite recent developments there is still an urgent need for ultra-small high-affinity intein tags for *in vitro* and *in vivo* approaches. To date, only very few in-cell applications of protein trans-splicing have been reported, all limited to C-terminal protein modifications. Here, we developed a strategy for covalent N-terminal intein-mediated protein labeling at (sub) nanomolar probe concentrations. Combined with a minimal synthetic lock-and-key element, the affinity between the intein fragments was increased more than 50-fold to 10 nM. Site-specific and efficient ‘traceless’ protein modification by high-affinity trans-splicing is demonstrated at nanomolar concentrations in living mammalian cells.

## Abstract 2

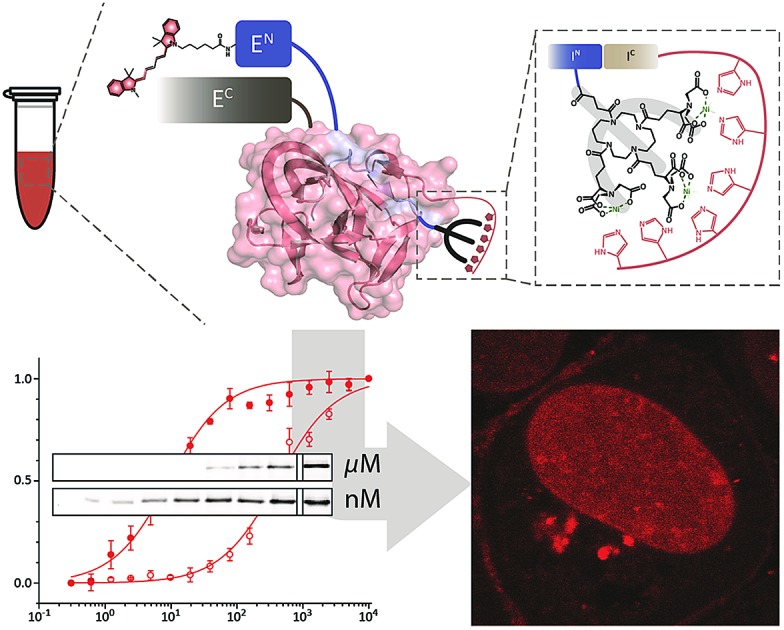
Using a minimal lock-and-key element the affinity between the intein fragments for N-terminal protein trans-splicing was significantly increased, allowing for site-specific, ‘traceless’ covalent protein labeling in living mammalian cells at nanomolar probe concentrations.

## Introduction

An eminent challenge in life sciences remains the site-specific protein modification with minimal perturbation for *in vitro* as well as *in vivo* analyses. Although the use of fluorescent proteins facilitated fundamental insight into protein function and cellular processes, their applicability is inherently limited due to their low photostability and quantum yield, slow maturation time, as well as large size.^1^ Hence, alternative approaches have been developed to introduce synthetic fluorescent probes with improved photo-physical properties by self-labeling proteins, *e.g.* SNAP/CLIP/Halo tags,^2^–^4^ or by enzymatic modifications *via* phosphopantetheinyl transferases, sortases, or lipoic acid ligases.^5^–^7^ Yet, these enzymes need to be supplied at high concentrations (1–100 μM) and the large fusion domains (>20 kDa) have adverse effects on the function, interaction, and trafficking of the target protein.

Besides these strategies, different chemoselective reactions and semi-synthetic techniques, such as native chemical ligation or intein-mediated protein splicing, have been established.^8^–^10^ Inteins are internal protein domains, which facilitate their own excision and thereby covalently fuse the flanking N- and C-terminal exteins, E^N^ and E^C^, in a self-processive manner. A very promising strategy for protein semi-synthesis is protein trans-splicing (PTS), which has been applied for segmental isotope labeling,^11^ protein backbone cyclization,^12^ cyclic peptide generation,^13^–^15^ protein immobilization,^9^,^16^,^17^ as well as for N- and C-terminal protein labeling.^18^–^21^ In PTS, the autocatalytic domain is naturally or artificially split into two fragments, I^N^ and I^C^, reconstituting the active intein complex.^22^

The excision process is virtually traceless, apart from a few flanking extein residues, required for efficient splicing. Whereas semi-synthetic PTS is widely applied *in vitro*, in-cell applications using split inteins are exclusively based on C-terminal protein modifications by trans-splicing.^23^–^26^ If the native C terminus is not accessible or essential for function, localization, or protein dynamics (*e.g.* lipid- and tail-anchored proteins,^27^ ubiquitin,^28^ and lamin A^29^), intracellular N-terminal protein modifications by PTS are indispensable. However, the large sizes of most split intein fragments with 100–130 amino acids (aa) for I^N^ and 35–50 aa for I^C^, also including high-affinity inteins, *e.g. Npu* DnaE^30^ or *Ssp* DnaE,^23^,^24^,^31^ compromise their accessibility by solid-phase peptide synthesis (SPPS).^32^,^33^

Several attempts were made to create short intein fragments for these semi-synthetic approaches.^20^,^34^ Despite that, the affinities in the micromolar range of very small intein fragments^18^ impede *in vivo* applications.

**Scheme 1:**
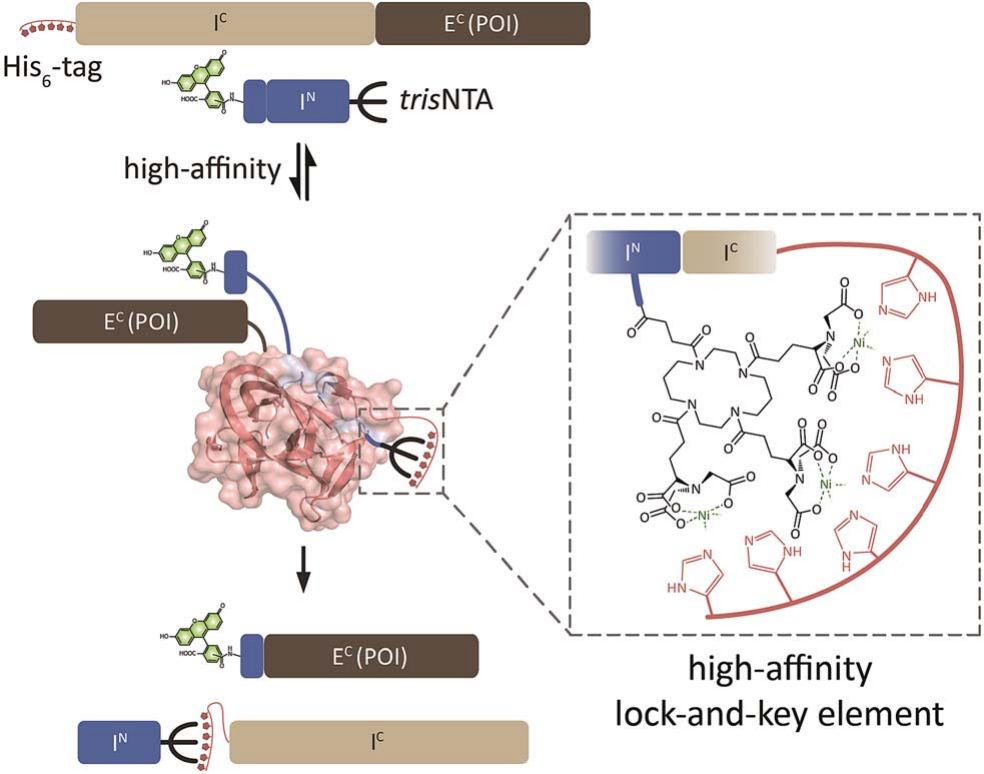
Protein trans-splicing guided by a minimalistic high-affinity interaction pair. Based on the high-affinity *tris*NTA/His-tag interaction, the intein fragments I^N^ and I^C^ react at nanomolar concentrations. PTS results in a ‘traceless’ N-terminal labeling of E^C^ (POI, protein of interest) with *e.g.* a molecular probe. *Ssp* DnaB: ; 1MI8.pdb.

Here, we adopted the multivalent chelator *tris-N*-nitrilotriacetic acid (*tris*NTA),^35^–^37^ which allows for site-specific and reversible recognition of His_6–10_-tagged proteins in the nanomolar range (*K*_D_ < 10 nM), even inside living cells.^38^,^39^ The *tris*NTA/His-tag system defines one of the smallest high-affinity recognition elements known to date. We developed a split intein system guided by this minimalistic interaction pair to promote N-terminal protein labeling at nanomolar concentrations by trans-splicing (Scheme 1). A related approach was realized with *E. coli* dihydrofolate reductase and its ligand trimethoprim.^40^ Nonetheless, this strategy suffers from a large fusion domain (25 kDa) and micromolar concentrations applied for labeling. Noteworthy, this affinity pair was utilized for extracellular labeling, whereas our high-affinity *tris*NTA/His-tag pair already demonstrated accurate labeling of proteins in living cells.^38^,^39^,^41^ Our approach combines this diminutive interaction pair with the smallest synthetically accessible I^N^ (11 aa) and a recombinantly expressed I^C^ (143 aa) of the artificially split DnaB M86 mini-intein from *Synechocystis* sp. PCC6803 (*Ssp* DnaB M86).^42^,^43^

## Results and discussion

### Protein trans-splicing directed by a high-affinity interaction pair

**Fig. 1:**
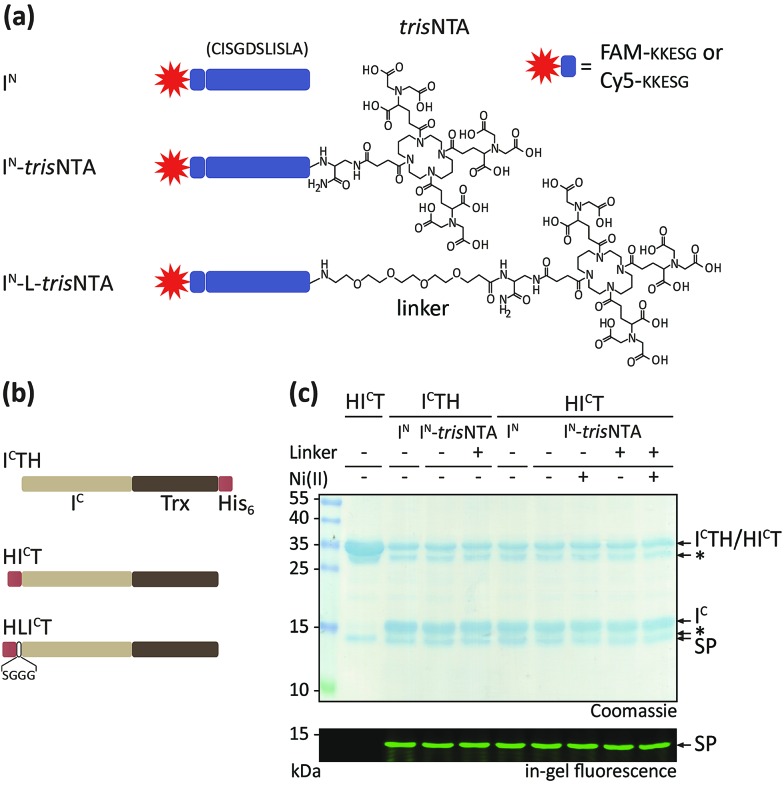
Semi-synthetic protein trans-splicing directed by a minimal high-affinity interaction pair. (a) Design of synthetic I^N^ fragments with five E^N^ residues (KKESG). TrisNTA was incorporated during Fmoc-based SPPS. 5(6)-Carboxyfluorescein (FAM) or Cy5 were used as fluorescent reporter. (b) Design of recombinant I^C^ with thioredoxin (Trx). N-terminal His_6_-tagged HI^C^T and HLI^C^T were used for interaction analyses with I^N^-*tris*NTA and I^N^-L-*tris*NTA. I^C^TH with a C-terminal His_6_-tag served as control. (c) PTS of I^C^TH/HI^C^T (10 μM) and FAM-labeled I^N^ (40 μM) yielded fluorescently labeled Trx (1 h, 20 °C). SP, splice product; *, impurity/premature cleaved intein.^20^,^44^

We generated a set of synthetic I^N^ and recombinant I^C^ fragments (Fig. 1a and b). I^N^ was prepared by Fmoc-based SPPS with five native E^N^ residues, KKESG, and either 5(6)-carboxyfluorescein or Cy5 as N-terminal fluorescent reporter. The multivalent chelator *tris*NTA was C-terminally introduced *via* diaminopropionic acid during SPPS (Fig. 1a and S1‡). Thioredoxin was chosen as a model protein and N-terminally fused with the I^C^ fragment of *Ssp* DnaB M86. In addition, the N terminus of I^C^ was equipped with a His_6_-tag and optionally a serine–glycine linker (SGGG, thereafter referred to as HI^C^T or HLI^C^T; Fig. 1b). For comparison, I^C^-Trx-His_6_ (I^C^TH) was used.^42^,^43^

We first investigated the trans-splicing reaction at micromolar concentrations typically used for *in vitro* experiments (Fig. 1c). After performing PTS, fluorescently labeled Trx and the excised I^C^ fragment were detected by SDS-PAGE in-gel fluorescence and Coomassie staining. Densitometric analysis of I^C^ conversion with His_6_-I^C^-Trx (HI^C^T; 76 ± 8%) and His_6_-I^C^-linker-Trx (HLI^C^T; 71 ± 10% for I^N^; Table S3‡) showed similar yields. These values are in accordance with data reported for I^C^TH and I^N^.^42^ >70% splice product (SP) formation was achieved for all constructs, with negligible formation of C-terminal by-products by premature cleavage of the intein. This phenomenon of self-induced C-terminal cleavage is known for the *Ssp* DnaB intein, but almost suppressed in the M86 mutant.^42^ SP formation was calculated as follows: [SP]/([SP] + [residual I^C^ fragment] + [cleaved I^C^]).^18^,^43^ The amount of premature cleaved intein^20^,^44^ was not included in SP determination. Our emphasis was not to prevent premature cleavage, but rather to increase the affinities of the intein fragments.

At 37 °C, PTS showed a decreased efficiency with up to 5-fold higher C-terminal cleavage.^42^ Interestingly, the Ser–Gly linker between the His_6_-tag and I^C^ (HLI^C^T) caused ∼10% less I^C^ conversion for I^N^-*tris*NTA and I^N^-L-*tris*NTA. The pseudo first-order rate of trans-splicing was *k*_PTS_ = (2.0 ± 0.6) × 10^–3^ s^–1^ corresponding to a *τ*_1/2_ = 14 ± 3 min at 20 °C for HI^C^T and I^N^. The rate is in perfect agreement with *k*_PTS_ = (2.5 ± 0.1) × 10^–3^ s^–1^ for I^C^TH under comparable conditions.^42^ All other constructs reacted similarly (Table S3‡). In conclusion, the presence of Ni-*tris*NTA and N-terminal His-tag had no effect on the trans-splicing efficiency and reaction kinetics.

### High-affinity interaction determined by fluorescence anisotropy and microscale thermophoresis

**Fig. 2:**
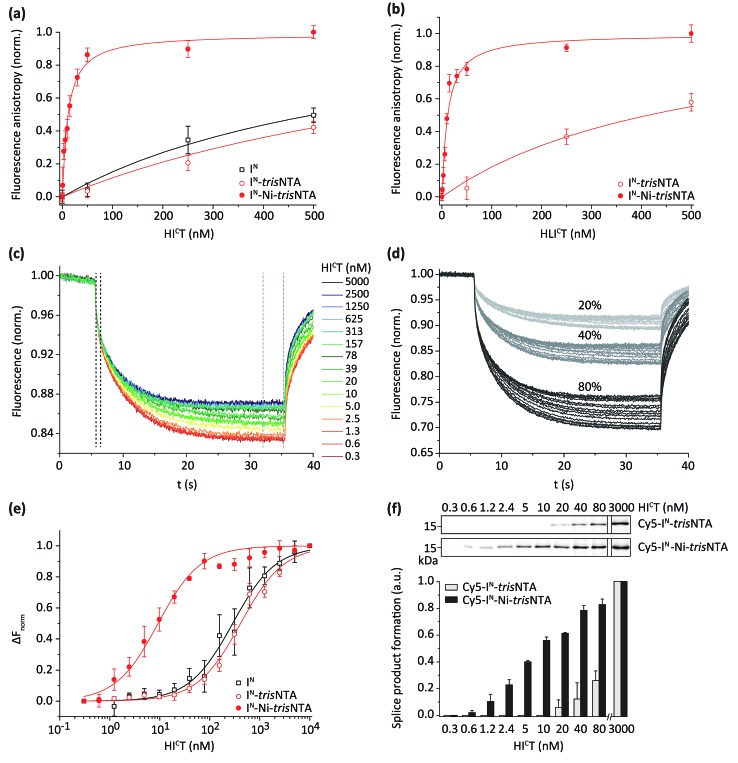
The *tris*NTA/His-tag pair triggers high-affinity interaction of intein fragments. (a) Interaction between FAM-I^N^ or FAM-I^N^-*tris*NTA (100 nM each) with either HI^C^T or HLI^C^T (b) was analysed by fluorescence anisotropy. A dramatic increase in affinity was observed for FAM-I^N^-Ni-*tris*NTA. Fluorescence anisotropy measurements were performed with splice-inactive I^C^.^42^,^43^ (c) Representative MST traces of Cy5-I^N^ (150 pM) with different concentrations of HI^C^T as indicated. (d) Variable MST power (20–80%) was applied to optimize the trace resolution. (e) The interaction between HI^C^T and Cy5-I^N^, Cy5-I^N^-*tris*NTA, or Cy5-I^N^-Ni-*tris*NTA (150 pM each) was determined by analysing changes in the MST signals. Interaction analysis was performed with splice-inactive I^C^.^42^,^43^ (f) Trans-splicing of Cy5-I^N^-*tris*NTA and Cy5-I^N^-Ni-*tris*NTA (1 nM) with increasing concentrations of HI^C^T (1 h, 20 °C). PTS was followed by SDS-PAGE in-gel fluorescence analysis. Error bars: S.D.

We determined the affinity of the intein fragments by fluorescence anisotropy (FA; Fig. 2a and b) and by microscale thermophoresis (MST)^45^,^46^ using the splice-inactive I^C(N154A, S+1A, H73A)^ mutant^42^,^43^ and 150 pM of Cy5-labeled I^N^ (Fig. 2c–e and S6‡). The MST experiments represent the first use of MST for affinity measurements performed on inteins.

By FA, an equilibrium dissociation constant *K*_D_ of 350 ± 60 nM was determined for the splice-inactive trans-splicing pair I^C^TH and I^N^. A similar *K*_D_ was observed for HI^C^T, whereas a negligible reduction of the affinity was detected for HLI^C^T. In the case of I^N^-*tris*NTA and I^N^-L-*tris*NTA, we observed slight decreased affinities for HI^C^T and HLI^C^T (Table 1).

**Table 1:** High-affinity intein interaction. Equilibrium dissociation constants *K*_D_ were determined using fluorescence anisotropy (FA) and microscale thermophoresis (MST)^*a*^

I^C^ fragment	I^N^ fragment	FA: *K*_D_ (nM)	MST: *K*_D_ (nM)
I^C^TH	I^N^	350 ± 60	n.d.
	I^N^-*tris*NTA	650 ± 60	n.d.
HI^C^T	I^N^	350 ± 90	285 ± 20
	I^N^-*tris*NTA	560 ± 65	410 ± 30
	I^N^-Ni-*tris*NTA	11 ± 1	9 ± 1
	I^N^-L-*tris*NTA	590 ± 50	500 ± 50
	I^N^-L-Ni-*tris*NTA	13 ± 1	8 ± 2
HLI^C^T	I^N^	410 ± 90	480 ± 90
	I^N^-*tris*NTA	510 ± 75	560 ± 40
	I^N^-Ni-*tris*NTA	10 ± 2	12 ± 3
	I^N^-L-*tris*NTA	610 ± 80	660 ± 70
	I^N^-L-Ni-*tris*NTA	9 ± 1	10 ± 4

^*a*^n.d. not determined.

In the case of I^N^-Ni-*tris*NTA, an impressive increase in affinity between the intein fragments was observed (*K*_D_ ∼ 10 nM). To further investigate high-affinity PTS, we employed MST. 150 pM of Cy5-I^N^ were titrated with increasing concentrations of HI^C^T (Fig. 2c). To better resolve the Cy5-fluorescence traces, diverse MST power was used (Fig. 2d). The fluorescence change Δ*F*_norm_ was deviated from *F*_hot_/*F*_cold_, whereat *F*_hot_ and *F*_cold_ are the mean fluorescence at the end of the measurement (Fig. 2c, light-grey dashed line) and 1 s after turning on the IR-laser (Fig. 2c, dark-grey dashed line).^47^ Δ*F*_norm_ of each MST trace is plotted against the respective I^C^ concentration to preserve a dose–response curve (Fig. 2e), from which the affinity can be derived. Notably, the *K*_D_ values, measured by two independent approaches, are very similar (Table 1). Importantly, the affinities between I^N^ and I^C^ were superimposed and promoted by the *tris*NTA/His-tag interaction.

We noted that the affinities of the *tris*NTA/His-tag modified intein pairs are in the same range as the naturally occurring *Npu* DnaE inteins for C-terminal modification,^30^ the rapamycin-triggered FKBP12-FRB,^48^,^49^ or the *E. coli* dihydrofolate reductase–trimethoprim interaction.^40^ However, in comparison, the ultra-small size of our interaction pair combined with the minimal synthetic I^N^ fragment (11 aa) makes this system the smallest nanomolar affinity intein described until now.

Next, we compared the trans-splicing reaction in the micromolar and nanomolar range (Fig. 2f). In line with the drastic increased affinity promoted by the *tris*NTA/His-tag interaction, trans-splicing was observed down to the sub-nanomolar range (0.6 nM). In the absence of the small lock-and-key pair, *e.g.* nickel-deficient *tris*NTA, PTS was not observed at low probe concentrations (Fig. 2f). The trans-splicing efficiencies and kinetics are summarized in the ESI (Table S3, Fig. S7–S9‡).

### Protein trans-splicing in the crowded cytosol of human cells

**Fig. 3:**
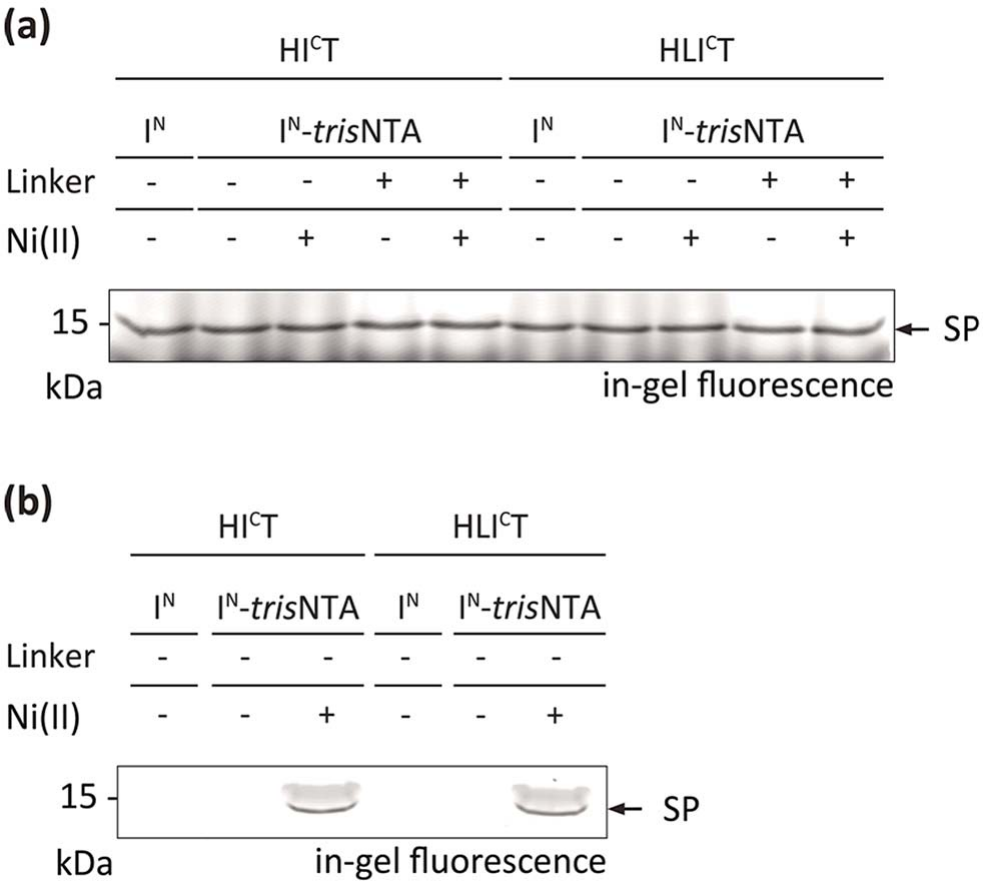
Protein trans-splicing in the cytosol of human cells promoted by the *tris*NTA/His-tag interaction. High-affinity protein labeling was performed with 10 μM of I^C^ and 40 μM of I^N^ fragments (a) as well as with 1 nM of I^C^ and 4 nM of I^N^ fragments (b) in HeLa cell lysate (20 mg mL^–1^). SP formation was analysed by SDS-PAGE in-gel fluorescence. Negative controls can be inferred from Fig. S10.‡

Encouraged by these results, we aimed at PTS in a crowded cellular environment. We analysed the high-affinity trans-splicing reaction in the cytosol of human cells (cell lysate). Using 10 μM of HI^C^T or HI^C^LT and 40 μM of the respective I^N^, a specific trans-splicing reaction and N-terminal modification of the target protein in cell lysate were detected (Fig. 3a). The trans-splicing efficiency was comparable to the *in vitro* results. A slightly lower SP efficiency was determined for I^N^-L-*tris*NTA, indicating that the Ser–Gly linker may affect the trans-splicing reaction. At nanomolar concentrations (1 nM), high-affinity PTS was exclusively observed with I^N^-Ni-*tris*NTA (Fig. 3b), whereas with I^N^ or I^N^-*tris*NTA no SP formation was detectable.

### Protein trans-splicing inside mammalian cells

**Fig. 4:**
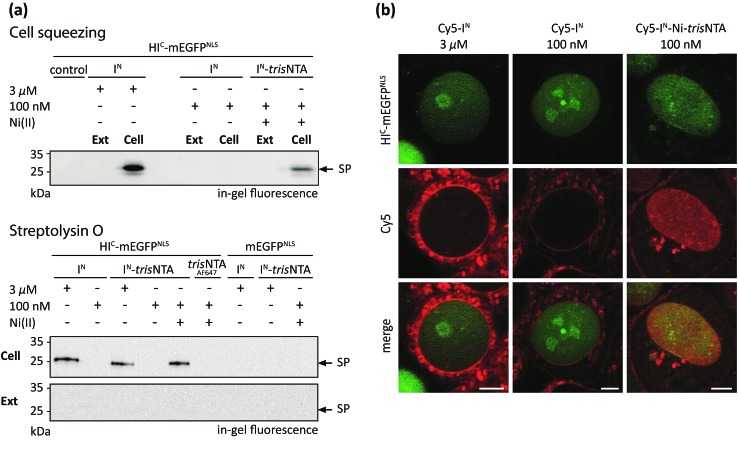
PTS in living mammalian cells. (a) Cy5-labeled I^N^ fragments were delivered into the cytosol of HeLa Kyoto cells transfected with HI^C^-mEGFP^NLS^ or mEGFP^NLS^*via* cell squeezing or SLO semi-permeabilization. At nanomolar concentrations, in-cell PTS was only observed with Cy5-I^N^-Ni-*tris*NTA (100 nM), whereas similar concentrations of Cy5-I^N^ or Cy5-I^N^-*tris*NTA showed no PTS reaction. In the absence of the high-affinity pair or without coordinated Ni(ii), 3 μM of Cy5-I^N^ or Cy5-I^N^-*tris*NTA was needed. For both permeabilization approaches, SP formation was detected only in cells (Cell) but not in the external medium (Ext). As a negative control for cell squeezing, 3 μM of Cy5-I^N^ in mEGFP^NLS^ transfected cells was used. Negative controls for SLO semi-permeabilization were performed with 3 μM of Cy5-I^N^ and Cy5-I^N^-*tris*NTA, as well as 100 nM of Cy5-I^N^-Ni-*tris*NTA in mEGFP^NLS^ transfected cells. (b) Cy5-labeled I^N^ probes were delivered into the cytosol of SLO semi-permeabilized HeLa Kyoto cells and imaged using CLSM. At 100 nM of Cy5-I^N^-Ni-*tris*NTA, co-localization with mEGFP^NLS^ was observed. In contrast, 100 nM of Cy5-I^N^ did not lead to specific labeling. Using 3 μM of Cy5-I^N^, low signal-to-background labeling was detected at the nucleus (see also Fig. S16‡). Scale bar: 5 μm.

The successful PTS reaction in cell lysate incited us to perform trans-splicing in living mammalian cells. To date, only four in-cell approaches of PTS have been reported, all limited to C-terminal modifications.^23^–^26^ To establish the first in-cell application of semi-synthetic PTS at the N terminus, we used HI^C^-mEGFP as a model protein. To achieve a distinct cellular distribution for visual readout, mEGFP was additionally equipped with nuclear localization sequences (NLS, HI^C^-mEGFP^NLS^). For rapid intracellular delivery of Cy5-labeled I^N^ fragments, two cell transduction methodologies were tested. The cell squeezing technique^50^ was applied for high-throughput delivery and subsequent ensemble measurements, whereas semi-permeabilization with streptolysin O (SLO) was additionally adapted to provide instantaneous excess to the cytosol for direct observation of intracellular processes by confocal laser-scanning microscopy (CLSM). Human cervical cancer (HeLa Kyoto) cells expressing HI^C^-mEGFP^NLS^ were transduced in the presence of various concentrations of Cy5-labeled I^N^ fragments by cell squeezing as well as SLO semi-permeabilization. In both cases, we first investigated the formation of covalently modified trans-splicing product (Cy5-mEGFP^NLS^) by SDS-PAGE in-gel-fluorescence analysis (Fig. 4a). At micromolar concentrations of Cy5-I^N^, we observed SP formation in squeezed as well as semi-permeabilized cells whereas nanomolar concentrations of Cy5-I^N^ failed to yield the trans-splicing product Cy5-mEGFP^NLS^. In contrast, 100 nM of Cy5-I^N^-Ni-*tris*NTA still resulted in SP formation, demonstrating that the trans-splicing reaction is boosted by the minimal lock-and-key recognition element even in living cells (Fig. 4a and b and S13 and S14‡). Notably, no SP was detected in cells expressing mEGFP^NLS^ lacking the HI^C^ part. Interestingly, comparable SP yields were obtained for Cy5-I^N^ and Cy5-I^N^-*tris*NTA at micromolar concentrations as well as for 100 nM of Cy5-I^N^-Ni-*tris*NTA, indicating that the chemical composition of the I^N^ fragments does not bias the trans-splicing reaction. Moreover, the guidance by the minimal *tris*NTA/His-tag interaction is essential for SP formation at nanomolar concentrations, since no SP formation was observed for Cy5-I^N^-*tris*NTA lacking Ni(ii). It is worth mentioning that no SP was detected in the external medium (Ext, Fig. 4a), confirming that high-affinity PTS occurred inside cells (Cell; Fig. 4a). Cytotoxic effects of Ni(ii) ions can be excluded due to the high complex formation constant of 10^–13^ M of Ni-NTA. As a result, free nickel ions only exist in the femtomolar range. These results demonstrate that the high-affinity interaction pair does not only boost PTS *in vitro* and in cell lysates, but even inside living cells.

The efficiency of trans-splicing in living cells was determined by immunoblotting analysis against GFP. The trans-splicing yield was determined to 2–8% for 3 μM of Cy5-I^N^ and 2% for 100 nM of Cy5-I^N^-Ni-*tris*NTA (Fig. S15‡). In contrast to *in vitro* analyses and trans-splicing in cell lysates, the efficiency in living mammalian cells dropped 10- to 40-fold. Although the *in vivo* PTS efficiency decreased, the high-affinity *tris*NTA/His-tag interaction still enables SP formation in the nanomolar range, as demonstrated by SDS-PAGE in-gel fluorescence analysis of transduced cells. These observations again highlight the benefit of the minimal lock-and-key recognition element for PTS.

Next, we aimed at visualization of SP formation in mammalian cells expressing HI^C^-mEGFP^NLS^. It is noteworthy that the fusion of the I^C^ sequence (∼150 aa) did not affect the nuclear localization of HI^C^-mEGFP^NLS^ (Fig. S12‡). Since cell squeezing dislocates mEGFP^NLS^ and squeezed cells need time for reattachment to the surface, this technique is not appropriate in this case. Therefore, we delivered the I^N^ fragments by SLO semi-permeabilization to provide instantaneous excess to the cytosol for direct observation of intracellular processes using confocal laser-scanning microscopy (CLSM) without a major time delay. Nuclear co-localization of the fluorescence signal for HI^C^-mEGFP^NLS^ and Cy5-I^N^-Ni-*tris*NTA provided evidence of site-specific and high-affinity PTS at nanomolar probe concentration in living cells (Fig. 4a and b and S15‡). As demonstrated by SDS-PAGE in-gel fluorescence, this is indicative of PTS at nanomolar concentrations (Fig. 4a). Here, only 100 nM of Cy5-I^N^-Ni-*tris*NTA was offered to the cells, since this concentration proved to be sufficient for PTS (Fig. 4a). In contrast, applying the same concentrations of unmodified Cy5-I^N^, no specific Cy5-labeling of nuclear-targeted mEGFP^NLS^ was detected. Although micromolar concentrations of Cy5-I^N^ led to trans-splicing and nuclear Cy5-staining (Fig. 4a and b), this signal was outshone by the extensive background fluorescence (Fig. 4b and S16‡). Here, the laser intensities were adjusted to the high concentrations of unmodified Cy5-I^N^ required for trans-splicing and hence suppress fluorescence signals emanating from nuclear localized Cy5-I^N^.

In summary, we demonstrated in-cell trans-splicing by using two different transduction techniques as well as two different readout methods. In all scenarios, the minimalistic lock-and-key element efficiently guided PTS in the nanomolar range, fully in line with the results achieved *in vitro* and in cell lysates.

## Conclusions

We have developed a high-affinity split intein system for ‘traceless’ tracing of proteins. In our study, the affinity of the artificial split *Ssp* DnaB M86 intein was synergistically interconnected with the ultra-small *tris*NTA/His-tag recognition element. As a result, covalent N-terminal protein labeling *in vitro* and subsequently, for the first time, in living mammalian cells is demonstrated. Comprehensive *in vitro* characterization by SDS-PAGE in-gel fluorescence analysis, fluorescence anisotropy as well as microscale thermophoresis measurements demonstrated a more than 50-fold increase in affinity for the cognate intein fragments by the *tris*NTA/His-tag interaction pair. Despite this, the intrinsic intein properties were not affected in regard to yields and kinetics of the SP formation. Moreover, the minimalistic lock-and-key element even allowed performing PTS at (sub) nanomolar concentrations *in vitro* as well as *ex vivo* (human cell lysates) with comparable results. Hence, the trans-splicing reaction with minimal probe concentration is pivotal for advanced imaging techniques. As a first *in vivo* proof-of-concept, the developed high-affinity I^N^-*tris*NTA fragments were delivered into mammalian cells *via* microfluidic cell squeezing as well as semi-permeabilization. At nanomolar concentrations, SP formation in living cells was only detected in the presence of the high-affinity interaction pair and corroborated by SDS-PAGE in-gel fluorescence analysis. Thus, we established the first in-cell application of semi-synthetic protein trans-splicing at the N terminus. Although the trans-splicing efficiency was largely affected inside cells, the trans-splicing reaction is still guided by our minimal interaction pair and thereby promoted *in vivo*. To highlight opportunities for further development, particularly with respect to in-cell applications, FRET/quencher pairs to follow the trans-splicing reaction in real-time are currently under investigation. Taking advantage of the precision in labeling, we are exploring this high-affinity ‘traceless’ labeling approach for single-molecule tracking and localization analysis in live cells. This high-affinity N-terminal protein modification should advance our understanding of cellular networks.

## Supplementary Material

Supplementary information
