# Supplementary material for: ‘Traceless’ tracing of proteins – high-affinity trans-splicing directed by a minimal interaction pair
Source: Chem Sci. 2015 Dec 21;7(4):2646–52. doi: 10.1039/c5sc02936h (PMC5477019; doi:10.1039/c5sc02936h)
Supplement: Supplementary file 1 [file SC-007-C5SC02936H-s001.pdf]

Electronic Supplementary Material (ESI) for Chemical Science.
This journal is © The Royal Society of Chemistry 2015

Supporting Information

’Traceless’ Tracing of Proteins – High-Affinity *Trans*-Splicing Directed by a Minimal Interaction Pair

Markus Braner,[a] Alina Kollmannsperger,[a] Ralph Wieneke,[a] and Robert Tampé*,[a]

[a] Institute of Biochemistry, Biocenter, and Cluster of Excellence – Macromolecular Complexes, Goethe-University Frankfurt, Max-von-Laue-Str. 9, D-60438 Frankfurt/M., Germany

Table of Contents for Supporting Information

Abbreviations....S3
General remarks....S3
Plasmid construction for expression in *E. coli*....S3
Protein expression and purification....S4
Plasmid construction for expression in mammalian cells....S4
Preparation of HeLa cell lysate....S5
Cell culture and transfection....S5
Cell squeezing....S5
Semi-permeabilization with streptolysin O....S6
Protein *trans*-splicing assay....S6
Fluorescence anisotropy....S6
Microscale thermophoresis....S7
Synthesis of Fmoc-Dap(*tris*NTA-O*t*Bu)-OH **3**....S7
Solid-phase peptide synthesis and Ni(II)-loading....S8

Tab. S1 RP-C_{18} HPLC gradients S9
Tab. S2 Theoretical and experimental molecular weights of synthetic I^N fragments S10
Tab. S3 I^C conversion, pseudo first-order rate constants, and splice product formation S10

Fig. S1 Synthesis of Fmoc-Dap(*tris*NTA-O*t*Bu)-OH **3** S11
Fig. S2 Analytical RP-C_{18} HPLC of Fmoc-Dap(*tris*NTA-O*t*Bu)-OH **3** S11
Fig. S3 MALDI-TOF MS analysis of NHS-*tris*NTA-O*t*Bu **2** and Fmoc-Dap(*tris*NTA-O*t*Bu)-OH **3** S12
Fig. S4 Analytical RP-C_{18} HPLC of synthetic I^N fragments and MALDI-TOF MS analysis S13
Fig. S5 MALDI-TOF MS analysis of FAM-labeled thioredoxin S14
Fig. S6 Microscale thermophoresis analysis of Cy5-labeled I^N fragments with HLI^CT S14
Fig. S7 Protein *trans*-splicing at nanomolar concentration S15
Fig. S8 Kinetics of splice product formation at nanomolar concentration S15
Fig. S9 Protein *trans*-splicing followed by in-gel fluorescence S16
Fig. S10 Protein *trans*-splicing in cell lysates S17
Fig. S11 Expression of HI^C-mEGFP^{NLS} S18
Fig. S12 Efficient targeting of HI^C-mEGFP^{NLS} to the nucleus S18
Fig. S13 In-cell protein *trans*-splicing analysis by SDS-PAGE in-gel fluorescence S19
Fig. S14 In-cell protein *trans*-splicing concentration screen of I^N S19
Fig. S15 In-cell protein *trans*-splicing efficiency S20
Fig. S16 Amplified fluorescence signal of Cy5-I^N S21

References S21

Abbreviations

aa, amino acid; ATP, adenosine triphosphate; Boc, *tert*-butoxycarbonyl; CLSM, confocal laser-scanning microscopy; COMU, (1-cyano-2-ethoxy-2-oxoethylidenaminooxy)dimethylamino morpholino-carbenium hexafluorophosphate; Dap, diaminopropionic acid; DIPEA, *N*,*N*-diisopropylethylamine; DMEM, Dulbecco's modified eagle medium; DMF, dimethylformamide; DMSO, dimethylsulfoxide; DTT, dithiothreitol; EDC, 1-ethyl-3-(3-dimethylamino-propyl)-carbodiimide hydrochloride; EDT, 1,2-ethanedithiol; EDTA, ethylenediaminetetraacetic acid; EtOAc, ethyl acetate; EtOH, ethanol; FAM, 5(6)-carboxyfluorescein; FCS, fetal calf serum; Fmoc, fluorenylmethoxycarbonyl; fw, forward; HBTU, *O*-(benzotriazol-1-yl)-*N*,*N*,*N'*,*N'*-tetramethyluronium hexafluorophosphate; HEPES, 4-(2-hydroxyethyl)-1-piperazineethanesulfonic acid; His_6, hexahistidine; HOBt, hydroxybenzotriazole hydrate; HPLC, high performance liquid chromatography; MALDI-TOF MS, matrix-assisted laser desorption/ionization – time of flight mass spectrometry; MBHA, methylbenzhydrylamine; MeCN, acetonitrile; mEGFP, monomeric enhanced green fluorescent protein; MeOH, methanol; Mmt, monomethoxytrityl; MST, microscale thermophoresis; NHS, *N*-hydroxysuccinimide; NLS, nuclear localization sequence; NMM, *N*-methyl morpholine; NMP, *N*-methyl-2-pyrrolidon; NTA, *N*-nitrilotriacetic acid; ODS, octadecylsilane; PBS, phosphate buffered solution; PCR, polymerase chain reaction; rev, reverse; RP, reverse phase; RT, room temperature; S.D., standard deviation; SDS-PAGE, sodium dodecyl sulfate polyacrylamide gel electrophoresis; SLO, streptolysin O; SP, splice product; SPPS, solid-phase peptide synthesis; TBTA, tris(benzyltriazolylmethyl)amine; *t*Bu, *tert*-butyl; *t*BuOH, *tert*-butanol; TCA, trichloroacetic acid; TFA, trifluoroacetic acid; TIPS, triisopropylsilane; TLC, thin-layer chromatography; Trx, thioredoxin.

General remarks

Unless otherwise specified standard protocols were used. All reagents were of the highest analytical grade, supplied by Acros Organics (Geel, Belgium), GE Healthcare (Munich, Germany), Roth (Karlsruhe, Germany), Sigma-Aldrich (Munich, Germany) or VWR (Darmstadt, Germany). Fmoc-protected amino acids and COMU were obtained from Iris Biotech (Marktredwitz, Germany) except Fmoc-L-propargylglycine and Fmoc-15-amino-4,7,10,13-tetraoxapentadecanoic acid from Polypeptide (Strassbourg, France). $Cy5\text{-}N_3$ was obtained from Lumiprobe (Hannover, Germany). AlexaFluor647 NHS-ester was obtainded from Invitrogen. All assays were performed at least in triplicate and standard deviations (S.D.) were calculated. All restriction enzymes, Phusion high-fidelity DNA polymerase, DpnI, and T4 DNA ligase were purchased from Thermo Scientific.

Plasmid construction for expression in *E. coli*

The expression plasmids pIT21 (encoding for I^C-Trx-His_6)[1] and pIT28 (encoding for splice-inactive $I^{C(N154A,\ S+1A,\ H73A)}$-Trx-$His_6$)[1,2] were kindly provided by Henning D. Mootz (University of Münster, Germany). I^C is the C-terminal intein fragment (aa 12-154) from *Synechocystis sp.* PCC6803 DnaB (*Ssp* DnaB) M86 mini-intein.

<u>Plasmid construction His_6-I^C-Trx (**HI^CT**).</u> pIT21 was amplified with the primer pair forward (fw) 5'-**TAA**GCTTATCGATGATAAGCTGTCAAACATGAGAATTCTTGAAGACGAAAGGGCC-3' (stop codon bold) and reverse

(rev) 5'-AGATCTGTCGACAGAGCCGGCCAGGTTAGCG-3' to remove the His_6-tag. After digestion with DpnI, the resulting PCR product was ligated to yield a plasmid coding for I^C-Trx (I^CT). Afterwards this construct was amplified by whole cycle PCR with the primer pair fw 5'-*CATCATCATCATCATCAT*GGCACTAGTAGCACAGGAAAAAGAGTTCC GATTAAAGATTTGTTAGG-3' (His_6 italic) and rev 5'-CTAGTGCC*ATGATGATGATGATGATG*CATGGTATATCTCCTTCTT AAAGTTAAACAAAATTATTTCTAG-3' (His_6 italic) to introduce an N-terminal His_6-tag. After digestion with DpnI, subsequent ligation resulted in a plasmid coding for HI^CT. The identical procedure was performed for splice-inactive HI^CT.

Plasmid construction His_6-SGGG-I^C-Trx (**HLI^CT**). A serine glycine linker was introduced with the primer pair fw 5'-CATCAT*AGCGGAGGCGGT*GGCACTAGTAGCACAGGAAAAAGAGTTCC-3' (SGGG italic) and rev 5'-GCTACTAGTGCC *ACCGCCTCCGCT*ATGATGATGATGATGATG-3' (SGGG italic). After digestion with DpnI, the resulting product was ligated to generate a plasmid coding for HLI^CT. Plasmid construction of splice-inactive HLI^CT was performed in the same manner.

Protein expression and purification

For protein expression the respective plasmid was transformed via heat-shock into *E. coli* BL21 (DE3) cells. Protein expression and purification was performed as previously reported.[3] After Ni(II)-NTA affinity chromatography, the pooled fractions were dialyzed against buffer containing 50 mM Tris, 300 mM NaCl, 1 μM EDTA, 10% (v/v) glycerol, 2 mM DTT, pH 7.0. Afterwards, the activity of Trx was tested with an insulin-based method.[4] Protein concentration was determined by using the calculated molecular extinction coefficient at 280 nm. Finally purified protein was shock-frozen in liquid nitrogen and stored at -80 °C until experiments were performed.

Plasmid construction for expression in mammalian cells

Plasmid construction NLS-mEGFP-NLS (**$mEGFP^{NLS}$**). A nuclear localization sequence (NLS) was fused to both ends of mEGFP by PCR with the primer pair fw 5´-GCGCGATATCACC**ATG***CCTAAGAAAAAGCGGAAGGTG* GTGAGCAAGGGCGAGGAGCTGTTC-3´ (EcoRV restriction site underlined, NLS italic, start codon bold) and (rev) 5´-GCGCCTCGAG**TTA***CACCTTCCGCTTTTTCTTAGGGCC*CTTGTACAGCTCGTCCATGCCGAGAGTG-3´ (XhoI restriction site underlined, NLS italic, stop codon bold). The resulting PCR product was digested using the indicated restriction enzymes. A following insertion into the pCDNA3.1(+) vector (Life technologies) resulted in a plasmid coding for nuclear-targeted mEGFP ($mEGFP^{NLS}$).

Plasmid construction His_6-I^C-mEGFP (**HI^C-mEGFP**). His-tagged intein (HI^C) was amplified with the primer pair fw 5´-GCGCGCGGTACCTACCATGCATCATCATCATCATCATGG-3´ (Acc65I restriction site underlined) and rev 5´-GCGCGCACCGGTCCTCCTCCGGATCCTTCAATACTGTTATG-3´ (AgeI restriction site underlined) and afterwards cloned into pCDNA3.1(+) containing mEGFP (see above), using the indicated restricted enzymes. This resulted in a plasmid coding for HI^C-mEGFP.

Plasmid construction His_6-I^C-NLS-mEGFP-NLS (**HI^C-$mEGFP^{NLS}$**). The plasmid coding for HI^C-$mEGFP^{NLS}$ was constructed using FX-cloning.[5] HI^C was amplified by PCR with the primer pair fw 5´-TATATAGCTCTTCT**ATG**CATCATCATCATC-3’

(LguI FD restriction site underlined, start codon bold) and rev 5´-TATATAGCTCTTCTTCCTCCGGATCC-3´ (LguI FD restriction site underlined). mEGFP^{NLS} was amplified by PCR with the primer pair fw 5´-TATATAGCTCTTCTGGACCTAAGAAAAAG-3´ (LguI FD restriction site underlined) and rev 5´-TATATAGCTCTTCTTGCTTACACCTTCCG-3´ (LguI FD restriction site underlined). Restriction and ligation was performed in an one-pot reaction using the plasmid pcDXC3MS (addgene), both amplified fragments, LguI FD and T4 DNA ligase for 30 min at 37 °C. Transformation was performed in *E. coli* MC1061 cells.

Preparation of HeLa cell lysate

HeLa cells were grown up to 80-90% confluence in DMEM containing 10% (v/v) FCS (Gibco) under standard cell culture conditions (37 °C, 5% CO_2), harvested, and centrifuged at 120 x g (5 min, 4 °C). After washing once with PBS, centrifugation was repeated. The resulting cell pellet was dounced for 6-10 min on ice. Afterwards, centrifugation at 180,000 x g for 30 min at 4 °C was performed to remove cell membranes. The supernatant was used as a whole-cell lysate.

Cell culture and transfection

HeLa Kyoto were maintained in DMEM with 4.5 g/L glucose (Gibco), supplied with 10% (v/v) FCS. For passaging, cells were washed with PBS (Sigma-Aldrich) and detached from the culture dish with 0.05% trypsin/0.02% EDTA/PBS (GE Healthcare). Cell lines were stored in a humidified tissue culture incubator at 37 °C and 5% CO_2. Mycoplasma contamination tests were carried out regularly, following the guidelines described.[6] Transient transfection was performed with Lipofectamine 2000 (Life technologies), following the manufacturer's instructions. For streptolysin O (SLO) permeabilization and in-cell *trans*-splicing, 2 x 10^4 cells per well were seeded into 8-well on cover glass II slides (Sarstedt) and transfected with 0.2 μg DNA per well. For cell squeezing experiments, 8 x 10^5 cells were seeded into 6-well cell culture plates (Greiner) and transfected with 2 μg DNA per well. After transfection, cells were incubated 12-48 h at 37 °C and 5% CO_2 until experiments were performed.

Cell squeezing

Squeezing was performed using a chip with constrictions of 7 μm in diameter and 10 μm in length (CellSqueeze 10-(7)x1, SQZbiotech). In all microfluidic experiments, a cell density of 1.5 x 10^6 cells/mL in 10% (v/v) FCS/PBS were squeezed through the chip at a pressure of 30 psi. Transduction was conducted at 4 °C to block cargo uptake by endocytosis. During cell squeezing, the following cargo concentrations were used: 100 nM of Ni-*tris*NTA^{AlexaFluor647}, 100 nM of Cy5-I^{N}-Ni-*tris*NTA, 100 nM or 3 μM of Cy5-I^{N} (all diluted in PBS). After squeezing, cells were incubated for 5 min at 4 °C to reseal the plasma membrane.[7] Squeezed cells were washed with PBS, followed by washing with DMEM containing 10% FCS (Sigma-Aldrich) to remove unspecifically bound Ni-*tris*NTA^{AlexaFluor647} from the cell surface. After washing, cells were incubated for 20 min at RT for the *trans*-splicing reaction before they were lysed

by addition of SDS loading buffer (125 mM Tris, 20% glycerol, 5% β-mercaptoethanol, 4% (w/v) SDS, 0.02% (w/v) bromophenol blue) and heating at 95 °C for 10 min.

Semi-permeabilization with streptolysin O

HeLa Kyoto cells were transiently transfected with HI^{C}-mEGFP^{NLS}, which is targeted to the nucleus (Fig. S12). After washing twice with HBSS (137 mM NaCl, 5.4 mM KCl, 0.25 mM Na_2HPO_4, 0.44 mM KH_2PO_4, 4.2 mM $NaHCO_3$, 0.1% w/v D-glucose, 30 mM HEPES pH 7.2), I^N was introduced to the cells by semi-permeabilization using streptolysin O (SLO) by incubation (30 min, 20 °C) with 20 ng/mL SLO, 10 mM ATP, 30 mM $MgCl_2$, and I^N (3 μM or 100 nM of Cy5-I^N, 3 μM or 100 nM of Cy5-I^N-*tris*NTA, 100 nM of Cy5-I^N-Ni-*tris*NTA) in HBSS. To remove unspecifically bound Cy5-I^N-Ni-*tris*NTA, cells were subsequently washed with PBS and 10 mM histidine/10 mM ATP/30 mM $MgCl_2$/PBS. Images were recorded using the CLSM TCS SP5 (Leica) with a Plan-Appochromat 63 x/1.4 Oil DIC objective. Sequential imaging was performed to avoid crosstalk. The following laser lines were used for excitation: 488 nm (argon laser) for mEGFP, 633 nm (helium-neon laser) for Cy5. For image analysis, ImageJ[8] was used in combination with Fiji.[9]

Protein *trans*-splicing assay

All splicing reactions were performed in splicing buffer (50 mM Tris, 300 mM NaCl, 1 μM EDTA, 1 mM DTT, 10% glycerol, pH 7.0). I^N and I^C fragments were mixed at the indicated concentrations in splicing buffer and incubated for 1 h at 20 °C. In the nanomolar range proteins and peptides were precipitated with 100% (w/v) TCA. Afterwards the reaction was quenched by addition of SDS loading buffer (125 mM Tris, 20% glycerol, 5% β-mercaptoethanol, 4% (w/v) SDS, 0.02% (w/v) bromophenol blue) and boiled to 95 °C for 10 min before applying onto a reducing SDS-PAGE (12%). After in-gel fluorescence was detected with a Typhoon 9400 Imager (GE Healthcare) at ex/em = 488/526 nm (5(6)-carboxyfluorescein) and ex/em = 630/670 nm (Cy5) or with an Image Quant LAS 4000 (GE Healthcare) at ex/em = 460/510 nm (5(6)-carboxyfluorescein) and ex/em = 630/670 nm (Cy5), the gels were stained with Coomassie brilliant blue. The intensities of the protein bands were densitometrically determined by using 'Scion Image' or 'ImageJ'.[8] The pseudo first-order rate of protein *trans*-splicing was fitted by $P = P_0\ (1 - \exp(-kt))$, where P is the percentage of splice product formation at time t, P_0 is the splicing yield and k the observed rate.

Fluorescence anisotropy

Fluorescence anisotropy was analysed using a FluoroLog (Horiba Yvon) at ex/em = 488/512 nm. FAM-labeled I^N fragment (0.1 μM) was incubated with splice-inactive $I^{C(N154A,\ S+1A,\ H73A)}$ fragment (0.001-10 μM). Background fluorescence anisotropy of a blank sample (0.1 μM FAM-labeled I^N fragment) was subtracted from the final values. The increase in fluorescence anisotropy was monitored over 1 h for each protein concentration and the K_D was calculated by plotting the normalized fluorescence anisotropy against the protein concentration. Fitting was done with the following equation:

$$y = \frac{(K_D + R_0 + L_0) - \sqrt{(K_D + R_0 + L_0)^2 - 4R_0L_0}}{2R_0} \cdot B_{max}$$

(Eq. 1)

K_D: equilibrium dissociation constant; R_0: initial concentration of protein; L_0: initial concentration of peptide; B_{max}: maximal number of binding sites.

Microscale thermophoresis

Microscale thermophoresis was carried out by the Monolith NT.115Pico (NanoTemper, Munich). Splice-inactive $I^{C(N154A,\ S+1A,\ H73A)}$ fragments were serially diluted from 5000 down to 0.31 nM and 150 pM of Cy5-labeled I^N fragment was added. 0.05% (v/v) Tween-20 and 50 μM EDTA was added to the buffer. The samples were incubated at 20 °C for 5-10 min before they were loaded into the standard treated capillaries. All samples were measured by standard protocols (15% laser intensity, 20-80% MST power, IR laser λ = 1480 nm). The normalized fluorescence F_{norm} is the parameter to quantify binding via MST by the ratio of F_{hot} (fluorescence after thermodiffusion) and F_{cold} (initial fluorescence).[10]

$$F_{norm} = \frac{F_{hot}}{F_{cold}}$$

(Eq. 2)

Changes in the fluorescent thermophoresis signals were plotted against the concentration of the serially diluted I^C.

$$[AB] = \frac{[A] + [B] + K_D - \sqrt{([A] + [B] + K_D)^2 - 4[AB]}}{2[B]}$$

(Eq. 3)

A, B: binding partner; AB: bound complex; K_D: equilibrium dissociation constant. K_D values were determined by using Eq. 3.

Synthesis of Fmoc-Dap(*tris*NTA-O*t*Bu)-OH 3

Synthesis of NHS-*tris*NTA-O*t*Bu **2**. Carboxy-*tris*NTA-O*t*Bu[11] **1** (1.0 eq., 0.643 mmol), EDC (2.0 eq., 1.286 mmol) and NHS (1.2 eq., 0.7716 mmol) were dissolved in 100 mL dry DMF, covered under argon and stirred for 18 h at RT (Fig. S1). DMF was removed under vacuum and the remaining slurry was suspended in 100 mL EtOAc followed by washing with water (2 x 100 mL) in a separation funnel. The organic phase was dried over Na_2SO_4 and concentrated under reduced pressure to yield 64% in an impure manner. The impure product **2** was used without further purification. TLC: R_f = 0.7 in EtOAc/EtOH (9:1). MALDI-TOF MS: m/z = 1658.96 $[M+Na]^+$ (Fig. S3a).

Fmoc-Dap(*tris*NTA-O*t*Bu)-OH **3**. Compound **2** (1.0 eq., 0.518 mmol), Fmoc-Dap-OH*HCl (0.94 eq, 0.487 mmol) and DIPEA (fourfold excess, 2.072 mmol) were dissolved in 50 mL dry DMF and stirred for 18 h at RT under argon atmosphere (Fig. S1). DMF was removed under vacuum and the remaining slurry was suspended in 280 mL EtOAc transferred in a separation funnel and washed with 0.1 N HCl (2 x 50 mL), with water (1 x 50 mL) and saturated aqueous NaCl (1 x 50 mL). The organic phase was dried over Na_2SO_4 concentrated under reduced pressure and

purified by preparative RP-C_{18} HPLC (Vydac Grace 218TP1022 Protein & Peptide C18 column, 250 x 46 mm, 10 μm) with Method 1 (Tab. S1, Fig. S2). The desired fractions were lyophilized and compound **3** was received as a white powder (70% isolated yield). MALDI-TOF MS: m/z = 1849.2 $[M+H]^+$, 1871.3 $[M+Na]^+$ (Fig. S3b).

Solid-phase peptide synthesis and Ni(II)-loading

All I^N fragments were synthesized via Fmoc-based SPPS on a Rink amide MBHA resin as a solid support by a Liberty microwave peptide synthesizer (CEM, Kamp-Lintford, Germany) with a standard protocol (54 W, 3 min, 75 °C; 50 °C for cysteine building blocks). The amino acid side-chains were protected as follows: Asp(*t*Bu), Cys(Mmt), Glu(*t*Bu), Lys(Boc), and Ser(*t*Bu). The coupling reactions with the Liberty microwave peptide synthesizer were performed by activating 0.2 M of Fmoc-protected amino acid with 0.5 M of HBTU and HOBt in the presence of 2 M DIPEA in NMP. A double coupling for all amino acids was performed. Removal of the Fmoc group was carried out with 20% piperidine in DMF. The manual coupling reactions were performed twice with 1.5 eq. of Fmoc-protected amino acid, fluorophore or Fmoc-Dap(*tris*NTA-O*t*Bu)-OH, 3.0 eq. COMU and 6.0 eq. DIPEA (0.5 M in DMF) for 2 h at RT. Cy5-N_3 was introduced via click chemistry with Fmoc-L-propargylglycine. Thereby 1.0 eq. alkyne (50 nmol) and 2.0 eq. Cy5-N_3 (100 nmol) were clicked in 400 μL freshly prepared click solution (0.1 M CuBr/0.1 M TBTA 1:2 in DMSO/*t*BuOH 3:1, containing 40% DMF) directly on the resin. The mixture was thoroughly shaken at 25 °C over night. The click reaction was performed twice. Manual coupling of 5(6)-carboxyfluorescein (FAM) was performed twice using 5.0 eq. FAM, 4.7 eq. HBTU and 6.0 eq. NMM (400 mM in DMF).

The cleavage of the peptides was achieved by an incubation with a cleavage cocktail containing TFA/H_2O/TIPS/EDT/phenol (95:1.25:1.25:1.25:1.25) for 2 h or TFA/H_2O/TIPS/thioanisole/phenol (90:2.5:2.5:2.5:2.5) for 1 h for Cy5-labeled peptides. Cleaved peptides were precipitated in ice-cold diethyl ether, centrifuged, washed with ice-cold diethyl ether, dissolved in *t*BuOH/H_2O (4:1), and finally lyophilized. The purification was performed by semi-preparative RP-C_{18} HPLC (PerfectSil C18 column 250 x 22 mm 300 ODS, 5 μm, MZ Analytical, Mainz, Germany) under acidic conditions (buffer A: 0.05% TFA (aq.); buffer B: MeCN + 0.05% TFA) with 4 mL/min. The products were confirmed by mass spectrometry analysis. The purity of the peptides was monitored by analytical RP-C_{18} HPLC (PerfectSil C18 column 250 x 10 mm 300 ODS, 5 μm, MZ Analytical, Mainz, Germany) and detection at 215 nm, 460 nm for FAM, or 646 nm for Cy5 (Fig. S3). The purified peptides were lyophilized and solubilized in 20 mM HEPES, pH 7.0. Afterwards a 50-fold excess of $NiCl_2$ was added and the mixture was incubated for 1 h at 25 °C in the dark. Purification of fluorophore-labeled I^N-Ni-*tris*NTA and I^N-L-Ni-*tris*NTA was performed by anion exchange chromatography (1 mL HiTrap Q HP, GE Healthcare). After washing with 5 column volumes 50 μM EDTA in 20 mM HEPES, pH 7.0, the compound was eluted with a gradient starting from 0 to 1 M NaCl in 20 mM HEPES, pH 7.0. Peptide concentrations were determined by UV/Vis spectroscopy (Cary 50 Bio UV/Vis Spectrophotometer, Varian, Palo Alto, USA) at λ = 492 nm for FAM (ε_{492} = 65,000 M^{-1} cm^{-1}) or at λ = 646 nm for Cy5 (ε_{646} = 250,000 M^{-1} cm^{-1}).

Tab. S1 RP-C_{18} HPLC gradients. Purification of Fmoc-Dap(*tris*NTA-O*t*Bu)-OH **3** was performed using Method 1. FAM- and Cy5-labeled peptides were purified by Method 2 and 3, respectively.

Method 1		
t (min)	H_2O/MeOH 40/60 (%)	MeOH (%)
0.0	0.0	100.0
4.0	0.0	100.0
4.1	18.0	82.0
18.0	100.0	0.0
18.1	100.0	0.0
23.0	100.0	0.0
23.1	0.0	100.0
28.0	0.0	100.0

Method 2		
t (min)	0.05% TFA (aq.) (%)	MeCN + 0.05% TFA (%)
0.0	95.0	5.0
5.0	95.0	5.0
37.0	59.0	41.0
42.0	0.0	100.0
47.0	0.0	100.0
55.0	95.0	5.0

Method 3		
t (min)	0.05% TFA (aq.) (%)	MeCN + 0.05% TFA (%)
0.0	95.0	5.0
35.0	0.0	100.0
45.0	0.0	100.0
50.0	95.0	5.0

Tab. S2 Theoretical and experimental molecular weights of synthetic I^N fragments. Molecular weights were determined by MALDI-TOF MS (positive mode).

Compound	Formula	MW_{calc} (Da)	MW_{obs} (Da)
FAM-I^N	$C_{88}H_{129}N_{19}O_{30}S$	1964.89	1966.20, 2005.06 $[M+K]^+$
FAM-I^N-*tris*NTA	$C_{132}H_{194}N_{28}O_{54}S$	3067.30	3068.59
FAM-I^N-L-*tris*NTA	$C_{143}H_{215}N_{29}O_{59}S$	3314.44	3314.46
Cy5-I^N	$C_{109}H_{172}N_{26}O_{27}S$	2309.26	2310.34, 2333.30 $[M+Na]^+$
Cy5-I^N-*tris*NTA	$C_{153}H_{237}N_{35}O_{51}S$	3412.67	3413.77
Cy5-I^N-L-*tris*NTA	$C_{164}H_{258}N_{36}O_{56}S$	3659.62	n.d.

[n.d.]: not determined.

Tab. S3 I^C conversion, pseudo first-order rate constants, and splice product formation. Parameters of PTS were determined at 10 μM (white) and 10 nM (highlighted in gray) of the indicated I^C fragments. The amount of premature cleaved intein was not included in I^C conversion and splice product yield.

Concentration	I^C fragment	I^N fragment	I^C conversion (%)	Splice product (%)	k (10^{-3} s^{-1})
10 μM	HI^CT	I^N			2.0 ± 0.6
		I^N-*tris*NTA	76 ± 8	86 ± 7	2.5 ± 0.3
		I^N-L-*tris*NTA			2.2 ± 0.5
	HLI^CT	I^N	71 ± 10		2.4 ± 0.3
		I^N-*tris*NTA	66 ± 7	82 ± 6	2.3 ± 0.7
		I^N-L-*tris*NTA			1.8 ± 0.7
10 nM	HI^CT	I^N-Ni-*tris*NTA	68 ± 15	50-80	2.2 ± 1.1
		I^N-L-Ni-*tris*NTA	67 ± 12		
	HLI^CT	I^N-Ni-*tris*NTA	71 ± 14	45-75	1.8 ± 0.9
		I^N-L-Ni-*tris*NTA	65 ± 17		

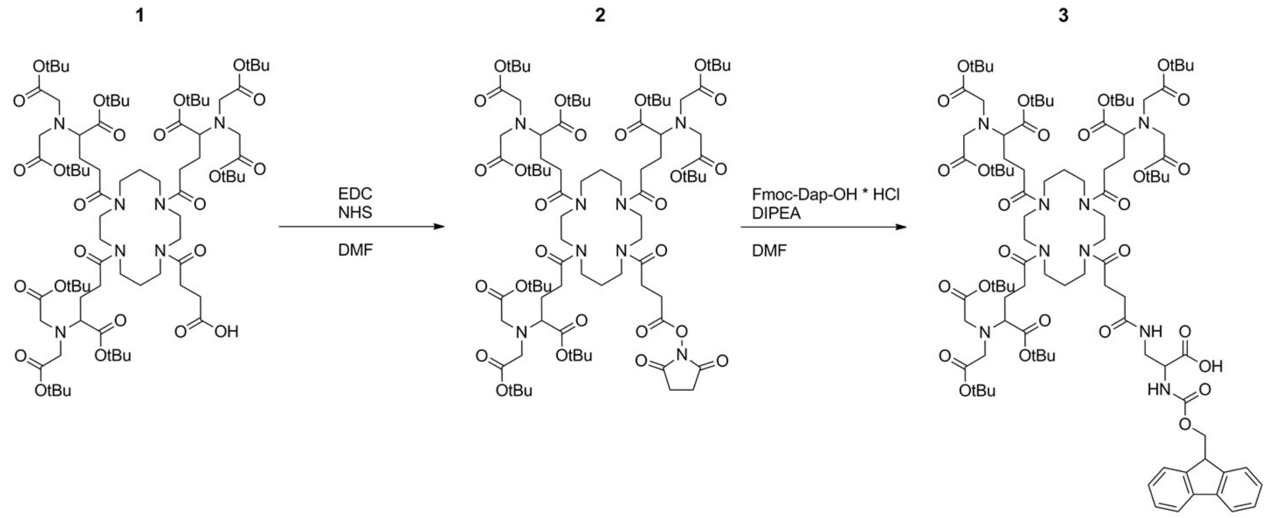

Fig. S1 Synthesis of Fmoc-Dap(*tris*NTA-O*t*Bu)-OH **3**. Carboxy-*tris*NTA-O*t*Bu **1** was synthesized as previously described.[11] NHS-*tris*NTA-O*t*Bu **2** was prepared by EDC/NHS activation in anhydrous DMF. The free primary amine of Fmoc-Dap-OH*HCl reacted with NHS in the presence of a fourfold excess of DIPEA to give Fmoc-Dap(*tris*NTA-O*t*Bu)-OH **3**.

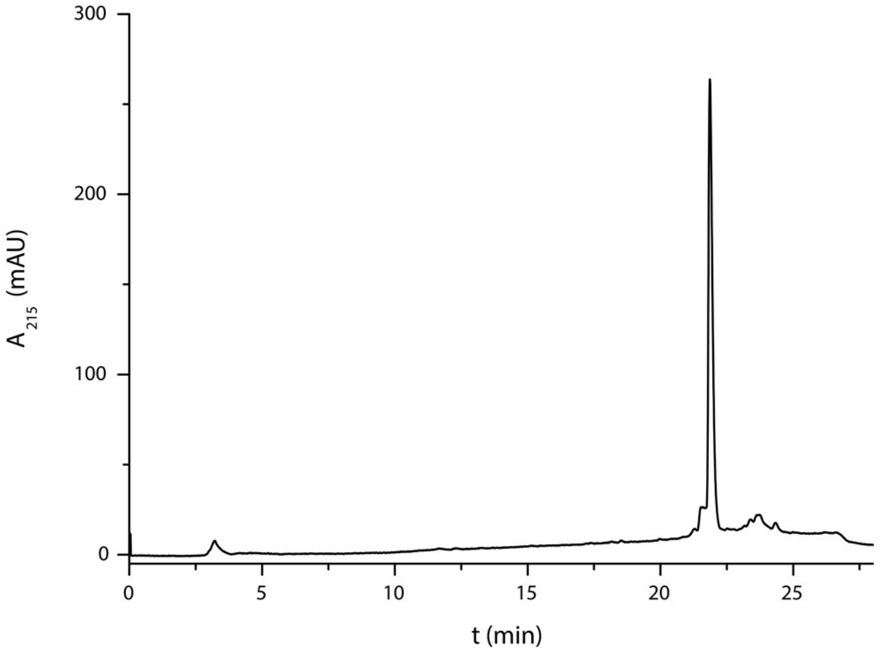

Fig. S2 Analytical RP-C_{18} HPLC of Fmoc-Dap(*tris*NTA-O*t*Bu)-OH **3.** Analysis was performed using a PerfectSil C18 column 250x10 mm 300 ODS, 5 μm (MZ Analytical, Mainz, Germany) with 1 mL/min and Method 1 (Tab. S1). Preparative purification was performed using a Vydac RP-C18 column 250 x 46 mm, 10 μm with 20 mL/min. Purity: > 90 %.

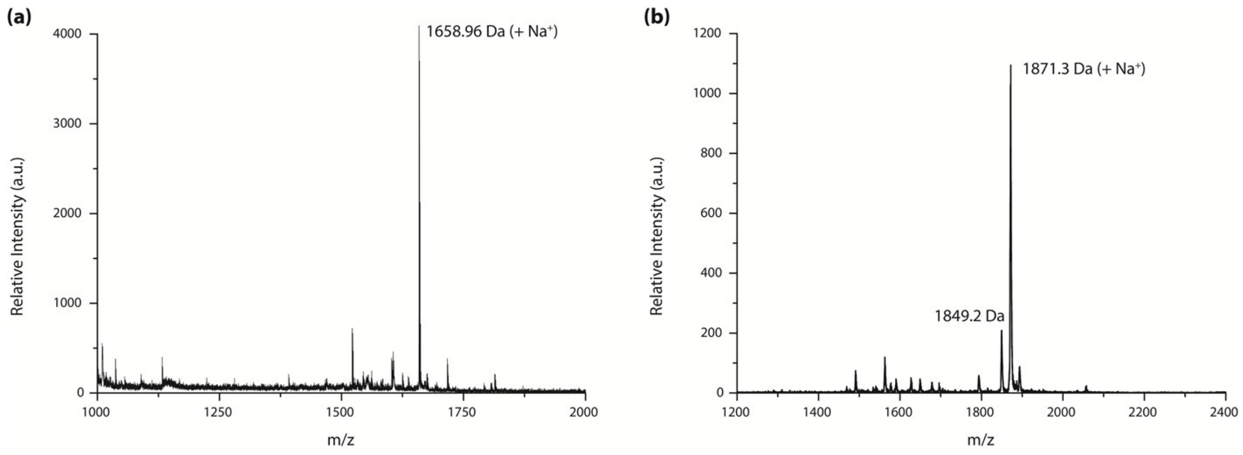

Fig. S3 MALDI-TOF MS analysis of NHS-*tris*NTA-O*t*Bu **2** and Fmoc-Dap(*tris*NTA-O*t*Bu)-OH **3**. (a) For NHS-*tris*NTA-O*t*Bu **2** a molecular weight of 1658.96 Da $[M+Na]^+$ was obtained. (b) A molecular weight of 1849.2 Da $[M+H]^+$ and 1871.3 Da $[M+Na]^+$ was observed for Fmoc-Dap(*tris*NTA-O*t*Bu)-OH **3**.

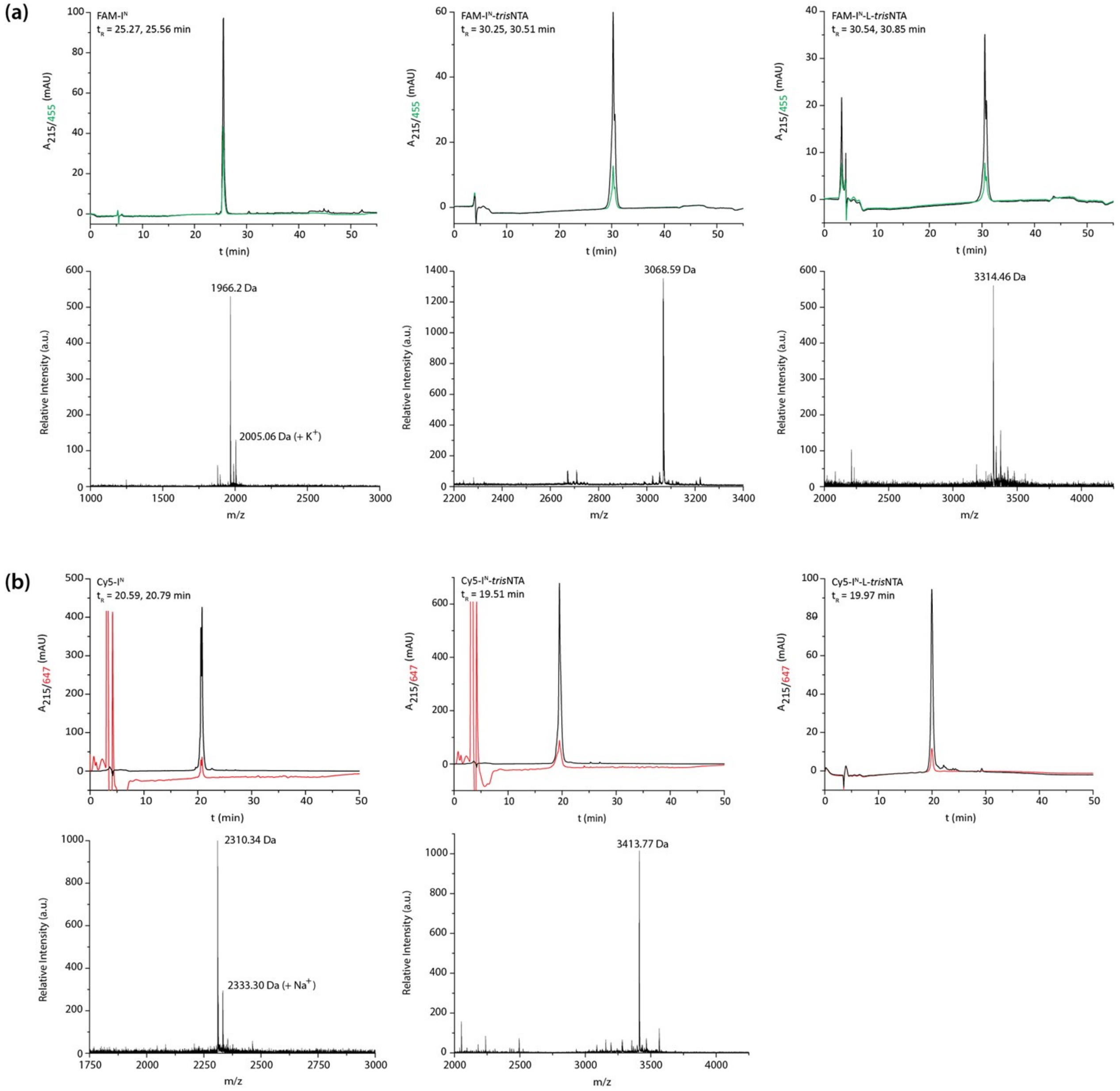

Fig. S4 Analytical RP-C_{18} HPLC of synthetic I^N fragments and MALDI-TOF MS analysis. (a) Purification and analysis of FAM-labeled I^N fragments (Method 2, Tab. S1) and (b) Cy5-labeled I^N fragments (Method 3, Tab. S1) were performed with PerfectSil C18 column 250 x 22 mm 300 ODS, 5 μm (MZ Analytical, Mainz, Germany) with 4 mL/min. Double peaks for FAM-labeled I^N (a) resulted from mixed isomers of FAM. Calculated and observed molecular weights are listed in Tab. S2.

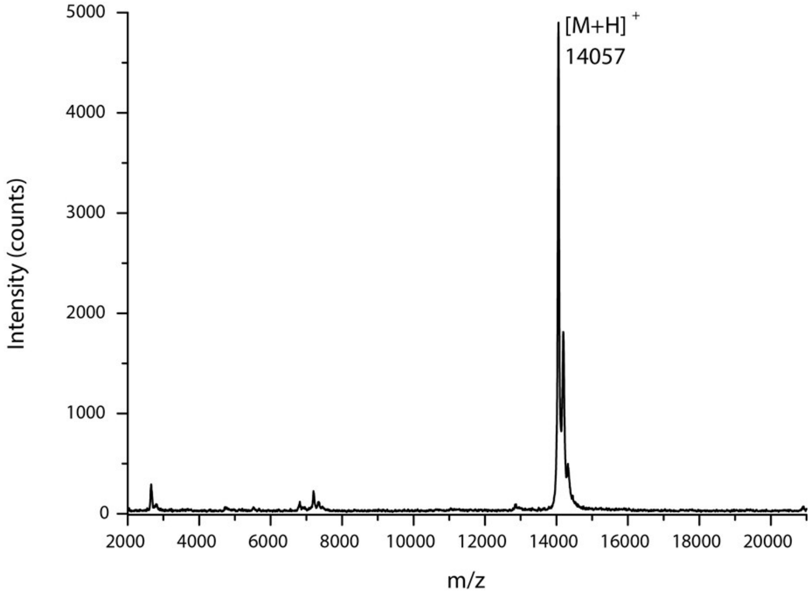

Fig. S5 MALDI-TOF MS analysis of FAM-labeled thioredoxin. The splice product (FAM-Trx) was purified by anion-exchange chromatography, dialyzed against ddH_2O and analysed by MALDI-TOF MS (20 μM final concentration). MW_{calc}: 14056.6 Da. MW_{obs}: 14057.0 Da.

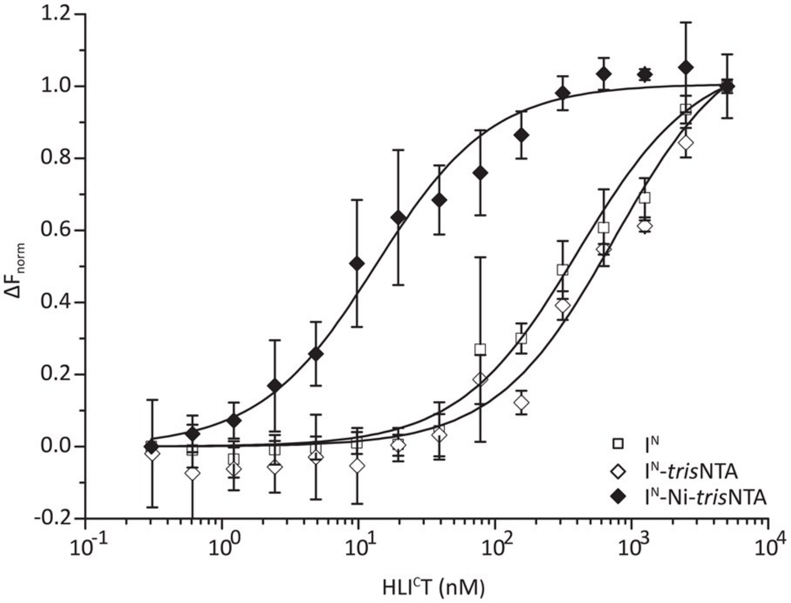

Fig. S6 Microscale thermophoresis analysis of Cy5-labeled I^N fragments with HLI^CT. Cy5-I^N, Cy5-I^N-*tris*NTA and Cy5-I^N-Ni-*tris*NTA (150 pM each) were titrated with different concentrations of splice-inactive HLI^CT. Changes in fluorescence were normalized (ΔF_{norm}). In case of Cy5-I^N-Ni-*tris*NTA, the affinity of the intein fragments was shifted to the sub-nanomolar range.

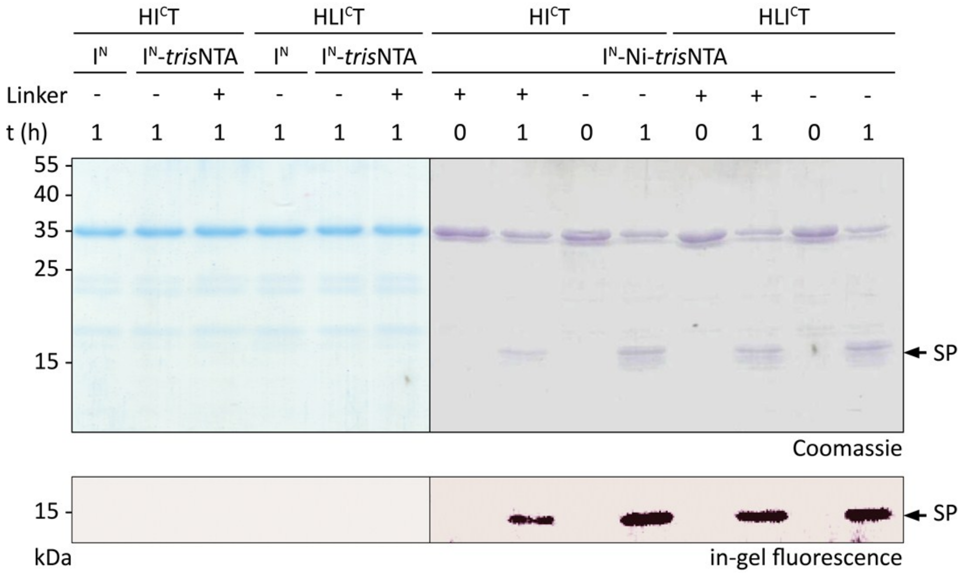

Fig. S7 Protein *trans*-splicing at nanomolar concentration. No SP formation was observed with I^N and I^N-*tris*NTA after 1 h at 20 °C. Only in case of I^N-Ni-*tris*NTA or I^N-L-Ni-*tris*NTA, SP was detectable after 1 h at 20 °C. All reactions were performed with 10 nM of HI^CT/HLI^CT and 40 nM of I^N fragments. Protein precipitation was done with 100% (w/v) TCA.

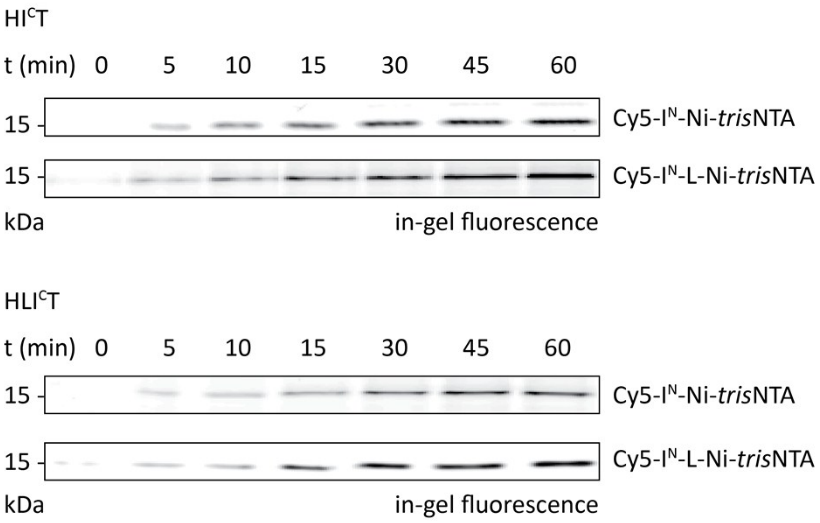

Fig. S8 Kinetics of splice product formation at nanomolar concentration. 10 nM of I^C and 40 nM of Cy5-I^N-Ni-*tris*NTA were incubated for 1 h at 20 °C. For both I^C constructs, nearly identical SP formation was detectable for the I^N fragments analyzed. Notably, with I^N-*tris*NTA or I^N-L-*tris*NTA no SP formation was observed. SP formation in the nanomolar and micromolar range was comparable, regarding to relative signal intensities. Due to the low sample amount, Coomassie staining was not possible.

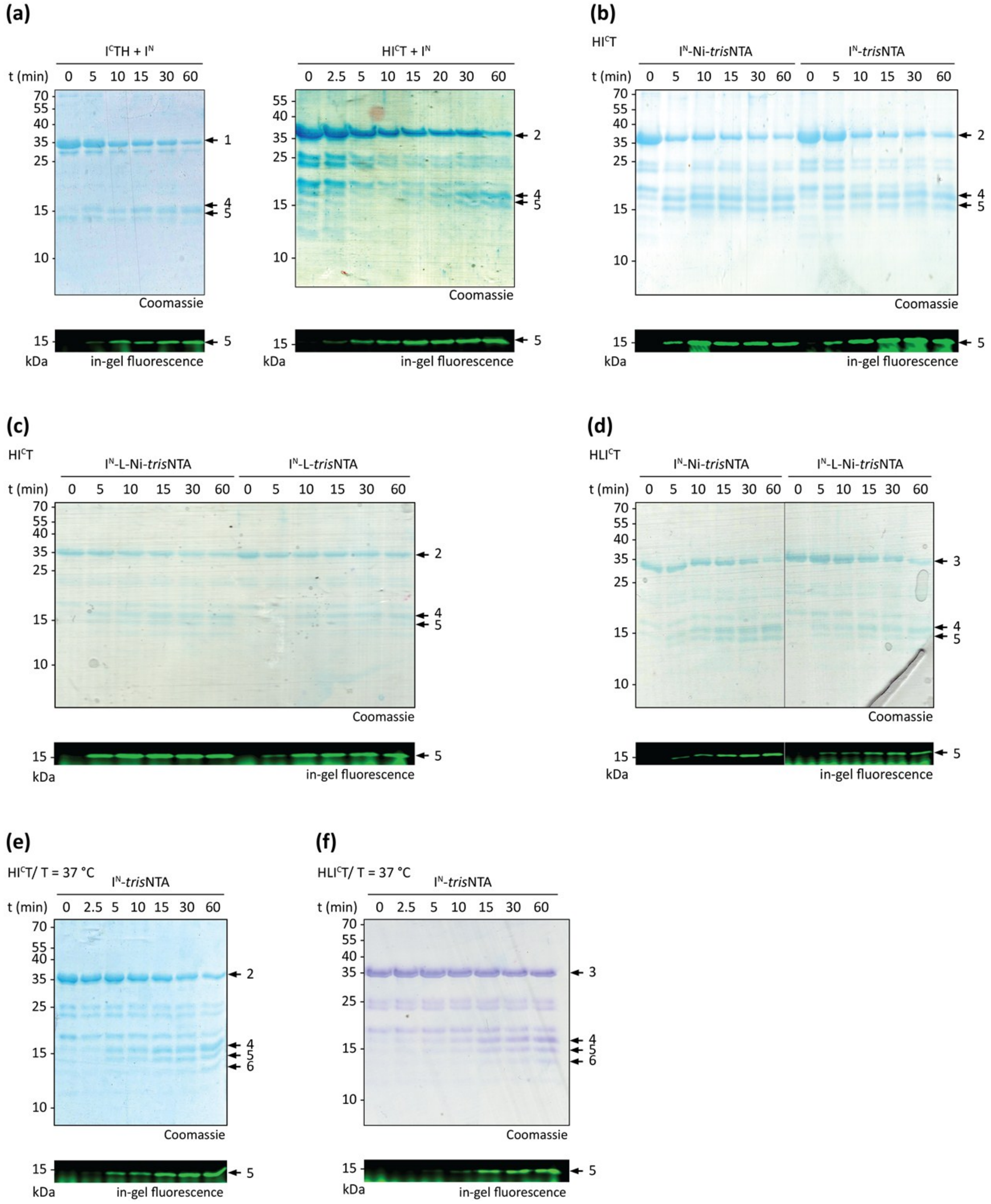

Fig. S9 Protein *trans*-splicing followed by in-gel fluorescence. (a) Comparison between I^{C}TH and HI^{C}T reacting with FAM-labeled I^{N}. Nearly identical I^{C} conversion was observed after 1 h at 20 °C. (b) The intein pairs of HI^{C}T/I^{N}-*tris*NTA and HI^{C}T/I^{N}-Ni-*tris*NTA offer slightly faster reaction kinetics than the previously reported I^{C}TH. (c) Using HI^{C}T/I^{N}-L-*tris*NTA or HI^{C}T/I^{N}-L-Ni-*tris*NTA, comparable SP formation, regarding non-modified I^{N} (a), was observed. (d) Up to 10% less I^{C} conversion of HLI^{C}T was detected in comparison to I^{C}TH and HI^{C}T. (e) and (f) When the *trans*-splicing reaction takes place at 37 °C, up to 5-fold increase in side products (C-terminal cleavage) was observed. All reactions were carried out with 10 μM of I^{C} and 40 μM of I^{N}. 1: I^{C}TH; 2: HI^{C}T; 3: HLI^{C}T; 4: I^{C}; 5: SP; 6: side product/premature cleaved intein.[1,12] Fluorescence signals below 5 correspond to the respective FAM-labeled I^{N} fragments.

1	cell lysate
2	cell lysate + 10 μM HI^CT
3	cell lysate + 40 μM FAM-I^N
4	cell lysate + 40 μM FAM-I^N-*tris*NTA
5	cell lysate + 40 μM FAM-I^N-Ni-*tris*NTA
6	cell lysate + 10 μM HI^CT/40 μM FAM-I^N

cell lysate: 20 mg/mL

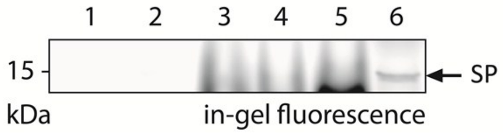

Fig. S10 Protein *trans*-splicing in cell lysates. HeLa cell lysate (1) with 10 μM HI^CT (2) or with 40 μM of the respective FAM-labeled I^N fragments (3-5) was used as negative control. High-affinity protein labeling was performed with 10 μM of HI^CT and 40 μM of FAM-I^N as positive control (6). SP formation was followed by SDS-PAGE in-gel fluorescence.

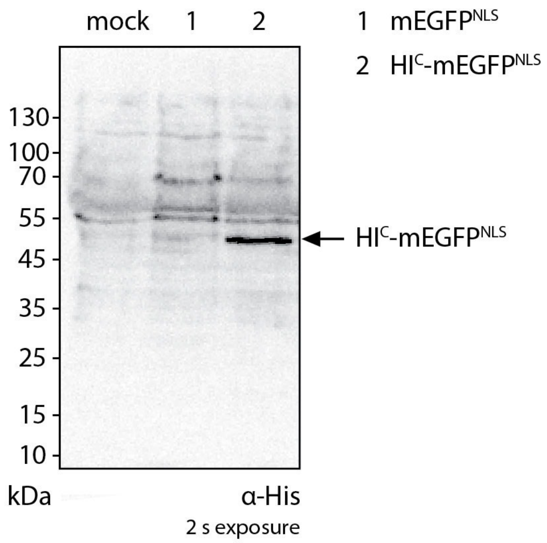

Fig. S11 Expression of HI^{C}-$mEGFP^{NLS}$. HeLa Kyoto cells, non-transfected (mock), transfected with $mEGFP^{NLS}$ (1) or HI^{C}-$mEGFP^{NLS}$ (2), were analysed by SDS-PAGE (12%) and immunoblotting with a primary mouse monoclonal anti-His antibody (Sigma-Aldrich) followed by incubation with a secondary HRP-conjugated anti-mouse antibody (Sigma-Aldrich). Exposure time: 2 s.

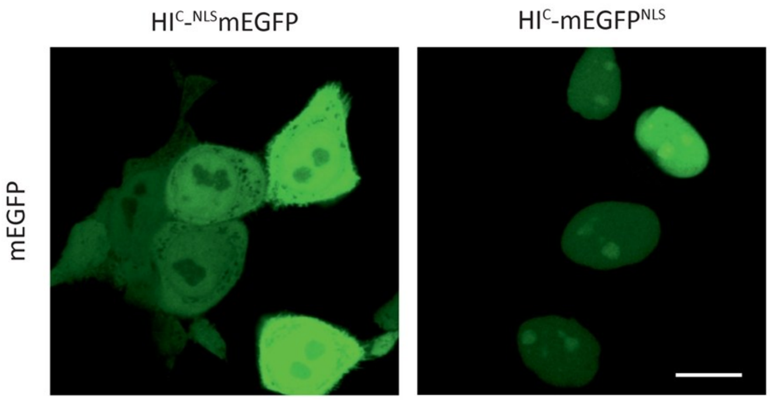

Fig. S12 Efficient nuclear targeting of HI^{C}-$mEGFP^{NLS}$. HeLa Kyoto cells transiently transfected with HI^{C}-^{NLS}mEGFP or HI^{C}-$mEGFP^{NLS}$ were imaged by CLSM. HI^{C}-^{NLS}mEGFP harboring only a single N-terminal nuclear location signal (NLS) was incompletely targeted to the nucleus with a significant portion of mEGFP remaining in the cytosol (left), whereas HI^{C}-$mEGFP^{NLS}$ containing an N- and C-terminal NLS sequence was efficiently recruited to the nucleus. Scale bar: 10 μm.

	transfection	cargo
1	mock	
2	mEGFP	
3	HI^{C}-mEGFP	
4	mock	100 nM Cy5-I^{N}-Ni-*tris*NTA
5	HI^{C}-mEGFP	3 μM Cy5-I^{N}
6	HI^{C}-mEGFP	100 nM Cy5-I^{N}
7	HI^{C}-mEGFP	100 nM Cy5-I^{N}-Ni-*tris*NTA

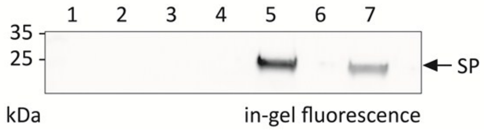

Fig. S13 In-cell protein *trans*-splicing analysis by SDS-PAGE in-gel fluorescence. HeLa Kyoto cells were transfected with the respective constructs. The cargos were delivered into the cytosol via cell squeezing.[7] Specific covalent N-terminal labeling of mEGFP was observed for 3 μM of Cy5-I^{N} (5), whereas no SP formation at nanomolar concentration was detectable (6). In the presence of Ni-*tris*NTA, SP formation was observed in the nanomolar range (7).

	transfection	cargo
1	untransfected	
2	mEGFP^{NLS}	
3	HI^{C}-mEGFP^{NLS}	
4	mEGFP^{NLS}	3 μM Cy5-I^{N}
5	HI^{C}-mEGFP^{NLS}	3 μM Cy5-I^{N}
6	HI^{C}-mEGFP^{NLS}	1 μM Cy5-I^{N}
7	HI^{C}-mEGFP^{NLS}	500 nM Cy5-I^{N}
8	HI^{C}-mEGFP^{NLS}	100 nM Cy5-I^{N}
9	mEGFP^{NLS}	100 nM Cy5-I^{N}-Ni-*tris*NTA
10	HI^{C}-mEGFP^{NLS}	100 nM Cy5-I^{N}-Ni-*tris*NTA

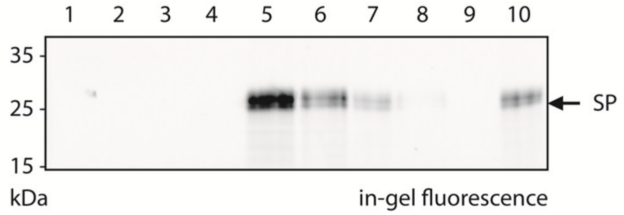

Fig. S14 In-cell protein *trans*-splicing concentration screen of I^{N}. HeLa Kyoto cells were transfected with either mEGFP^{NLS} or HI^{C}-mEGFP^{NLS}. The synthetic cargos were delivered into the cytosol via cell squeezing.[7] Protein *trans*-splicing was performed with concentrations ranging from 3 μM down to 100 nM of non-modified Cy5-I^{N} and with 100 nM of Cy5-I^{N}-Ni-*tris*NTA for high-affinity interaction. Subsequently, SP formation (Cy5-mEGFP^{NLS}) was detected via in-gel fluorescence. Based on densitometric analysis nearly similar SP formation was observed for 1 μM of Cy5-I^{N} and 100 nM of Cy5-I^{N}-Ni-*tris*NTA.

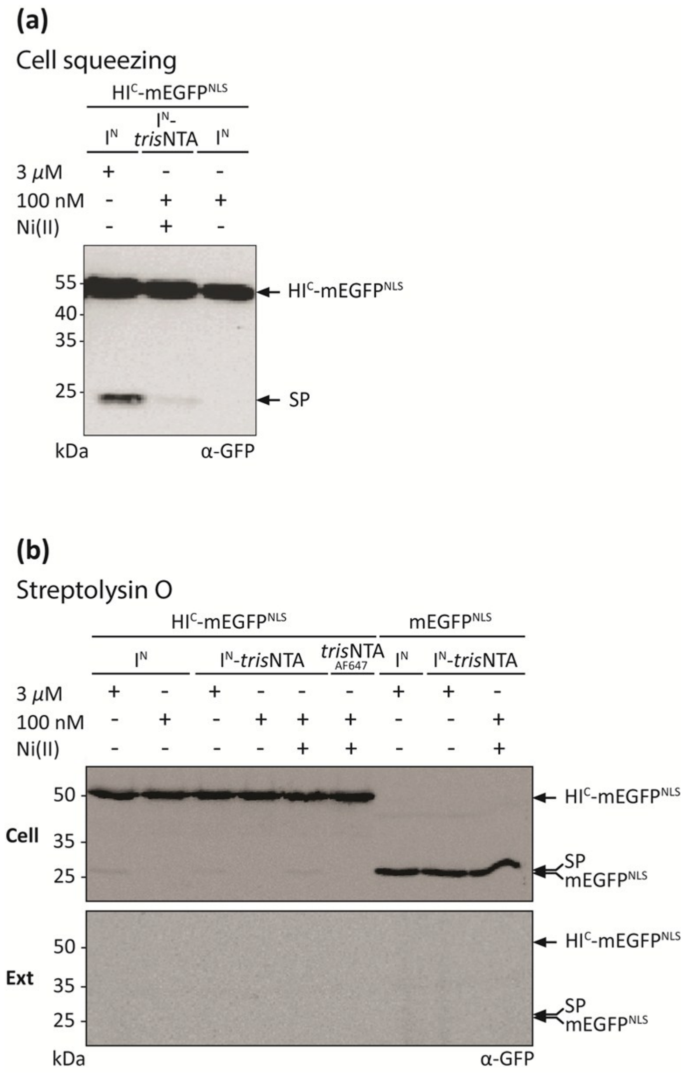

Fig. S15 In-cell protein *trans*-splicing efficiency. (a) Cy5-labeled I^N fragments were delivered into HeLa Kyoto cells transfected with HI^C-$mEGFP^{NLS}$ *via* cell squeezing.[7] (b) Cy5-labeled I^N fragments were delivered by SLO semi-permeabilization into the cytosol of HeLa Kyoto cells transfected with HI^C-$mEGFP^{NLS}$ or $mEGFP^{NLS}$. Efficiency of SP formation was determined by immunoblotting analysis against GFP. SP formation was exclusively detected in $2\ 10^6$ cells (Cell) but not in the external medium (Ext; volume adapted to C, $2\ 10^6$ cells per sample). Thereby, 2-8% *trans*-splicing product was observed for 3 μM of Cy5-I^N and 3 μM of Cy5-I^N-*tris*NTA, and 2% for 100 nM of Cy5-I^N-Ni-*tris*NTA with HI^C-$mEGFP^{NLS}$. In contrast, applying 100 nM of Cy5-I^N no SP was formed. As negative control for SLO semi-permeabilized cells $mEGFP^{NLS}$ with the indicated I^N fragments was used.

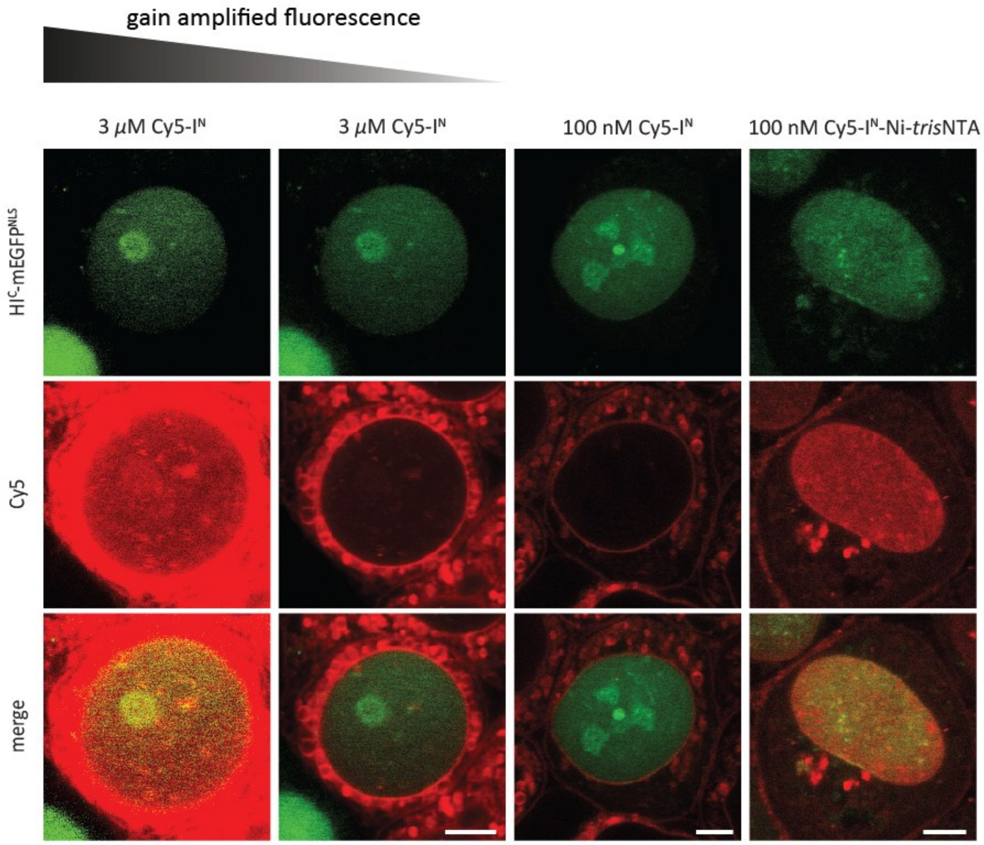

Fig. S16 Amplified fluorescence signal of Cy5-I^{N}. HeLa Kyoto cells were transiently transfected with HI^{C}-mEGFP^{NLS}, which is targeted to the nucleus (green). Two days after transfection, Cy5-labeled I^{N} was transduced into the cytosol (red). Because of the micromolar concentration of Cy5-I^{N} (3 μM), high background fluorescence was observed (left column). Therefore, the fluorescence intensity was artifically decreased by a factor of 5 (second column, see also Fig. 4b) for better visualization. Scale bar: 5 μm.

References

1 J. H. Appleby-Tagoe, I. V. Thiel, Y. Wang, Y. Wang, H. D. Mootz and X. Q. Liu, *J. Biol. Chem.*, 2011, **286**, 34440-34447.

2 A. Wasmuth, C. Ludwig and H. D. Mootz, *Bioorg. Med. Chem.*, 2013, **21**, 3495-3503.

3 C. Ludwig, M. Pfeiff, U. Linne and H. D. Mootz, *Angew. Chem. Int. Ed. Engl.*, 2006, **45**, 5218-5221.

4 A. Holmgren, *J. Biol. Chem.*, 1979, **254**, 9627-9632.

5 E. R. Geertsma and R. Dutzler, *Biochemistry*, 2011, **50**, 3272-3278.

6 C. C. Uphoff and H. G. Drexler, *Methods Mol. Biol.*, 2005, **290**, 13-23.

7 A. Sharei, J. Zoldan, A. Adamo, W. Y. Sim, N. Cho, E. Jackson, S. Mao, S. Schneider, M. J. Han, A. Lytton-Jean, P. A. Basto, S. Jhunjhunwala, J. Lee, D. A. Heller, J. W. Kang, G. C. Hartoularos, K. S. Kim, D. G. Anderson, R. Langer and K. F. Jensen, *Proc. Natl. Acad. Sci. U. S. A.*, 2013, **110**, 2082-2087.

8 V. Girish and A. Vijayalakshmi, *Indian J. Cancer*, 2004, **41**.

9 K. W. Eliceiri, M. R. Berthold, I. G. Goldberg, L. Ibanez, B. S. Manjunath, M. E. Martone, R. F. Murphy, H. Peng, A. L. Plant, B. Roysam, N. Stuurman, J. R. Swedlow, P. Tomancak and A. E. Carpenter, *Nat. Methods*, 2012, **9**, 697-710.

10 S. A. Seidel, P. M. Dijkman, W. A. Lea, G. van den Bogaart, M. Jerabek-Willemsen, A. Lazic, J. S. Joseph, P. Srinivasan, P. Baaske, A. Simeonov, I. Katritch, F. A. Melo, J. E. Ladbury, G. Schreiber, A. Watts, D. Braun and S. Duhr, *Methods*, 2013, **59**, 301-315.

11 S. Lata, A. Reichel, R. Brock, R. Tampé and J. Piehler, *J. Am. Chem. Soc.*, 2005, **127**, 10205-10215.

12 A. S. Aranko, J. S. Oeemig, D. Zhou, T. Kajander, A. Wlodawer and H. Iwai, *Mol. Biosyst.*, 2014, **10**, 1023-1034.
